# Mapping Morphology-Dependent Stability of Gold Nanostars in Immune Cells Using Hyperspectral Imaging

**DOI:** 10.1021/acs.analchem.6c03010

**Published:** 2026-06-18

**Authors:** Lakhvir Singh, Ngoc Nhu Vu, Elizabeth A. Bullard, Erin M. Stout, Samuel Mabbott, Alex J. Walsh

**Affiliations:** Department of Biomedical Engineering, 14736 Texas A&M University, College Station, Texas 77843, United States

## Abstract

Gold nanoparticles (AuNPs) are widely applied in nanomedicine, cellular and tissue biology, nanoscopy, photothermal therapy, and a range of diagnostic and clinical technologies. Among them, gold nanostars (AuNSs) have emerged as particularly promising due to their highly tunable optical and chemical properties. However, like other nanostructures, the stability of AuNSs remains a key challenge, especially within complex cellular microenvironments. Here, wide-field hyperspectral microscopy is evaluated for the real-time characterization of the morphology-dependent stability of AuNS formulations in immune-cell microenvironments. A computationally efficient image processing pipeline extracts statistical features from reflectance images, enabling the real-time analysis of hyperspectral data. UMAP-based visualization of spectral data revealed distinct, time- and formulation-dependent spectral shifts, with smaller seed volume formulations (larger overall diameter) for AuNSs exhibiting rapid destabilization and aggregation in THP-1 cells. In contrast, larger seed volume formulations (smaller overall diameter) for AuNS demonstrated enhanced colloidal stability and spectral uniformity. Compared to conventional ensemble measurements, hyperspectral reflectance measurements provided a rapid and resource-efficient approach that enabled macroscale imaging while retaining the spectral detail necessary to resolve AuNS transformations. Overall, the hyperspectral microscopy techniques presented here provide a label-free, high-throughput platform for evaluating AuNS stability and biocompatibility, with strong potential to guide the rational design of AuNSs for immunotherapeutic and diagnostic applications.

## Graphical abstract

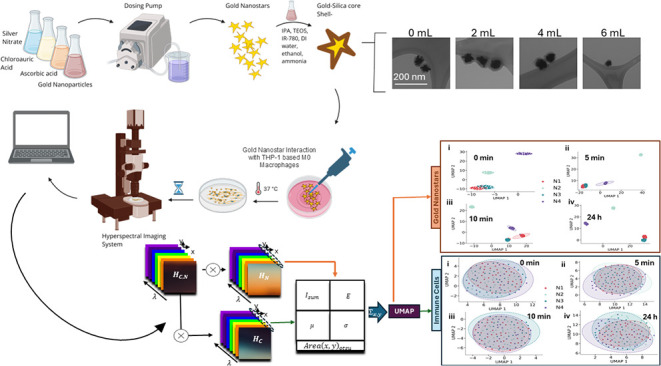

## Introduction

Nanoparticles (NPs) have become integral to modern science and technology, particularly in biomedical applications where their unique properties are harnessed for diagnostics, therapeutics, and imaging.^1^ Among these, gold nanostars (AuNS) represent a class of nanoparticles with highly tunable optical and chemical characteristics, making them exceptional candidates for targeted drug delivery, photothermal therapies, and bioimaging.^2^ The star-like morphology of AuNS enhances their optical properties through sharp tips that amplify local electromagnetic fields, enabling enhanced surface plasmon resonance (SPR) and scattering effects.^3^ These features allow for applications in both therapeutic and diagnostic modalities.^4^ However, despite their versatility, AuNSs, like many nanoparticles, are often unstable in complex biological environments.^5^ Their interactions with cellular components, particularly within immune-cell microenvironments, can significantly alter their structural integrity, functional performance, and biocompatibility.^6^

Nanoparticle stability is a critical parameter that governs their functionality and safety in biological systems.^7^ The term stability encompasses the preservation of size, shape, surface chemistry, and aggregation state, all of which can be influenced by factors such as ionic strength, protein adsorption, and cellular uptake.^8^ For instance, in immune-cell microenvironments, the high protein content and enzymatic activity can destabilize nanoparticles, leading to aggregation or surface modifications.^9–11^ These changes not only compromise the intended functionality of nanoparticles but can also result in cytotoxicity, limiting their utility in biomedical applications. Understanding and quantifying the stability of AuNS in such environments are thus imperative for optimizing their design and ensuring their safe deployment.

The nanoparticle can also play a pivotal role in determining their stability and interactions within biological systems.^12,13^ For AuNSs, the sharpness of the tips and overall dimensions influence their surface plasmon resonance, cellular uptake, and protein adsorption.^12,14^ Smaller nanoparticles tend to exhibit higher surface energy, making them more prone to aggregation, while larger nanoparticles may have reduced cellular penetration or altered biodistribution.^12^ These size-dependent behaviors are particularly relevant in immune-cell microenvironments, where the interaction of nanoparticles with macrophages, dendritic cells, and other immune cells can dictate their fate and function.^13^

Hyperspectral microscopy has emerged as a powerful tool for analyzing nanoparticles in complex biological media.^15,16^ This technique integrates spatial and spectral data, capturing reflectance spectra at the pixel level, to provide insights into nanoparticle stability, aggregation, and interactions. Unlike traditional methods, hyperspectral imaging enables real-time tracking of nanoparticles, allowing researchers to observe how they respond to environmental changes and interact with cells over time. For example, hyperspectral microscopy can distinguish between aggregated and dispersed nanoparticles based on their spectral signatures, providing a quantitative measure of aggregation state.^15,16^ Furthermore, by integrating machine learning techniques such as support vector machines (SVM) and spectral angle mapping (SAM) with spectral data, nanoparticles can be classified with high accuracy, even in noisy or complex data sets.^15^

A key consideration in translating optical imaging techniques from research settings to broader biomedical applications is accessibility. Wide-field and macroscale imaging approaches offer practical advantages in throughput and cost relative to confocal or multiphoton systems, and recent advances in affordable and compact implementations of optical modalities including reflectance imaging, fluorescence spectroscopy, and fluorescence lifetime imaging have expanded the range of measurements achievable outside of specialized facilities.^17–21^ The hyperspectral sensing framework presented here is designed with these considerations in mind, leveraging a wide-field, computationally efficient approach that prioritizes scalability and resource efficiency.

The purpose of this study was to establish a scalable and computationally efficient hyperspectral sensing framework for investigating morphology-dependent interactions between AuNS and immune cells. The morphologies of the AuNSs were modulated by varying the seed volume during synthesis. We employed a three-dimensional cross-correlation technique to map the nanoparticle–cell interaction dynamics of silica-coated AuNS at varying interaction times. By leveraging this computationally efficient method, we extract spectral-spatial metrics from reflectance hypercube and infer aggregation state, plasmonic coupling, and nanoparticle-cell interaction dynamics across formulations and time points. The findings from the work advance the broader field of nanomedicine by establishing design principles that link nanostar geometry and surface chemistry to colloidal stability and optical behavior in immune-cell microenvironments, directly informing the rational development of AuNS-based diagnostic and therapeutic platforms.

## Methods

### Materials

Gold­(III) chloride trihydrate (product no. 520918), hydrochloric acid (product no. 320331), and ascorbic acid (product no. A5960), silver nitrate (product no. S6506), ammonium hydroxide (product no. 221228, NH_3_), isopropanol (product no. 278475), tetraethyl orthosilicate (product no. 131903), and anhydrous trisodium citrate (product no. S1804) were purchased from Sigma-Aldrich and used for nanoparticle synthesis. Two hundred proof ethanol was obtained from Lab Alley LLC. THP-1 monocytes were obtained from the American Type Culture Collection (ATCC) and cultured in RPMI-1640 medium (catalog no. 11875135, Thermo Fisher Scientific) was supplemented with 10% fetal bovine serum (FBS, MT35010CV, Fisher Scientific). T-25 flasks (FB012935, FisherBrand) and 35 mm glass-bottom imaging dishes (Mattek, P35G-1.5-14-C) were used for cell culture and imaging. Ultrapure deionized water was used for all aqueous preparations as previously described.^22^

### Synthesis of AuNP Seed Suspension

Gold nanoparticle seeds were prepared by a citrate reduction. Briefly, 1.25 mL of 10 mM was added to 100 mL of deionized water in a round-bottomed flask equipped with a reflux condenser and brought to boiling under constant stirring. After the solution reached boiling, 1.3 mL of 1% trisodium citrate solution was added, and the mixture was boiled for an additional 15 min. During this period, the solution changed from clear to dark blue, then purple, and finally wine red, consistent with AuNP formation. The seed suspension was cooled to room temperature and washed by centrifugation at 5000 rpm for 30 min before nanostar growth, and the AuNP seed suspension was adjusted to an optical density of approximately 0.4 at 520 nm.

### Synthesis of Gold Nanostar Formulations

Gold nanostars were synthesized using the seed-mediated chemistry of a previously validated study,^22^ with manual reagent addition rather than automated pump delivery. Synthesis was carried out in a 250 mL Erlenmeyer flask containing a magnetic stir bar (0.7 × 3.5 mm) on a stir plate operating at 500 rpm. An acidic gold stock solution was prepared by mixing auric acid (0.25 mM) with 1 M HCl at a volumetric ratio of 1000:1. Under continuous stirring, 50 mL of the acidic solution was added to the reaction vessel, followed by AuNP seed suspension at 0, 2, 4, or 6 mL to generate the four formulations N1, N2, N3, and N4, respectively. After 1 min of mixing, 2 mL of 25 mM ascorbic acid and 2 mL of 0.5 mM silver nitrate were added simultaneously. The reaction proceeded for 2 min, after which the AuNS suspension was transferred to 50 mL centrifuge tubes and centrifuged at 5000 rpm for 30 min. The supernatant was discarded, the pellet was resuspended in 20 mL of deionized water, and the suspension was filtered through a 0.2 μm cellulose membrane (Whatman). Purified AuNS were stored at 4 °C until use.

### Silica Overcoating of AuNS

Silica overcoating was performed using the phase-2 encapsulation strategy previously described.^22^ Under continuous stirring, 35 mL of ethanol and 3 mL of aqueous were added to a fresh 250 mL Erlenmeyer flask and mixed for 5 min at 700 rpm. Next, 3 mL of AuNS suspension adjusted to OD = 0.4 was added and stirred for an additional 5 min (OD was calculated at the peak wavelength of the UV vis spectra for each sample). Subsequently, 85 mL of a silica precursor solution was added, and the mixture was stirred for 20 min while protected from light. The precursor stock contained 720 mL IPA, 9.6 mL TEOS, 72 mL deionized water. After shell growth, the reaction mixture was distributed into six 50 mL centrifuge tubes, adjusted to 50 mL with ethanol, and centrifuged at 14,000 rpm for 20 min. The pellets were combined and redispersed in 1 mL ethanol, then washed size times (4× with 200-proof ethanol and 2× with deionized water). Final silica-coated AuNS were dispersed in 2 mL deionized water and stored at 4 °C until use.

### Cell Culture and Differentiation

THP-1 monocytes were cultured in a culture medium prepared by adding 10% FBS added to RPMI-1640 Medium. THP-1 cells were maintained in T-25 flasks which were vertically rested in an incubator set at 37 °C with 5% CO_2_ and 95% humidified air. Media was exchanged twice a week by replacing 2 mL of the suspended cell-media mixture with a fresh culture medium. The cells were passaged once in 2 weeks, maintaining their viability above 85%. For imaging experiments, approximately 2,00,000 THP-1 cells were seeded into each 35 mm glass-bottom dish in 2 mL of culture medium. Differentiation to an M0 macrophage-like phenotype was induced by the addition of 20 μL phorbol 12-myristate 13-acetate (PMA) to each dish yielding a final concentration of 10 ng/mL. After 48 h, the differentiated THP-1-derived macrophages were adherent and used for AuNS exposure experiments.

### AuNS Exposure Protocol and Hyperspectral Imaging Timeline

Before nanoparticle exposure, the culture medium was removed, and the cells were washed once with PBS. A 2 mL aliquot of AuNS suspension was then added to each dish, and the cells were incubated with AuNS for 30 min at 37 °C. After incubation, the AuNS suspension was aspirated and the dishes were washed once with 2 mL PBS to remove excess particles. Two dishes were prepared for each formulation. Samples were imaged at 0 min, 5 min, 10 min, and 24 h. The 0 min time point refers to the first image acquired immediately after aspiration and washing. After washing, 2 mL of culture medium was added to the cells prior to imaging. After data collection at the acute time points (0, 5, and 10 min), the dishes were transferred to the incubator and maintained for 24 h, after which they were immediately imaged.

### Wide-Field Hyperspectral Reflectance Imaging

Hyperspectral imaging was performed using a custom-designed and built hyperspectral imaging system (HySAF) with 30 spectral bands, equally spaced at a 10 nm interval, between 440 to 730 nm.^23^ The cell-AuNS samples were imaged at 0 min (immediately after exposure), 5 min, 10 min, and 24 h, after AuNS addition. Images were acquired from two regions of interest (ROIs) near the centers of each imaging dish. Prior to sample acquisition, calibration images were captured by using a 99% reflective target and a dark background (black plastic optical blocker) to correct for system reflectance. Subsequently, images of 2 mL of each of the four AuNS formulations were collected in imaging dishes at two ROIs per sample.

### Transmission Electron Microscopy (TEM)

Transmission electron microscopy (TEM) was performed on silica-coated AuNS samples using a JEOL 1200 electron microscope. Samples were briefly sonicated for 30 s, and ∼7 μL of suspension was deposited onto a carbon film-coated copper grid (Ted Pella, Inc.). Each deposited aliquot was allowed to adsorb for 10 min, after which excess liquid was wicked away with filter paper. This loading step was repeated four times to ensure sufficient particle density for imaging. TEM images were analyzed using ImageJ^24^ to estimate particle size, morphology, and silica shell thickness. Size measurements were performed on a minimum of *n* = 20 particles per formulation, with each particle measured three times to assess reproducibility.

### UV–vis Spectroscopy

UV–vis absorbance measurements were performed by loading 200 μL of sample into a transparent 96-well plate (Thermo Fisher Scientific) and acquiring spectra from 350 to 1000 at 5 nm spectral resolution using a Tecan Infinite 200 Pro microplate reader. All measurements were performed in triplicate. Spectra were normalized to the minimum and maximum absorbance values within the measured wavelength range to enable direct comparison across formulations and time points. UV–vis characterization was performed for both bare and silica-coated AuNS prior to macrophage exposure to confirm successful silica coating and to document the seed volume-dependent shift in the surface plasmon resonance (SPR) peak. The optical stability of Si-AuNS was assessed by monitoring SPR peak position and spectral shape over 24 h in deionized water, PBS, and RPMI-1640 supplemented with 10% FBS prior to cell exposure. Additionally, spectra were acquired from aliquots collected from the imaging dishes at each time point (0 min, 5 min, 10 min, and 24 h) during macrophage interaction experiments to track nanoparticle optical changes upon cellular exposure.

### DLS and ζ-Potential Characterization

For DLS measurements, 100 μL of sonicated sample was diluted in 900 μL of deionized water and loaded into a disposable folded capillary cell (Malvern, DTS1070). Measurements were performed at 25 °C using a Malvern Zetasizer Nano ZS (Malvern Panalytical, Worcestershire, UK) equipped with a 633 nm He–Ne laser at a fixed backscatter detection angle of 173°. Size distributions were analyzed using the General Purpose (Normal Resolution) algorithm with the number of runs determined automatically by the instrument software. All samples were measured in triplicate. The polydispersity index (PDI) was recorded alongside the intensity-weighted mean hydrodynamic diameter (*Z*-average) as a measure of size distribution uniformity; samples with PDI < 0.2 were considered monodisperse. For ζ-potential measurements, 750 μL of the diluted sample was transferred to the same capillary cell and analyzed using the Smoluchowski approximation at 25 °C. DLS, PDI, and ζ-potential characterization was performed for both bare and silica-coated AuNS prior to macrophage exposure. The colloidal stability of Si-AuNS was assessed by monitoring hydrodynamic diameter, PDI, and ζ-potential over 24 h in deionized water, PBS, and RPMI-1640 supplemented with 10% FBS prior to cell exposure. Additionally, measurements were obtained from aliquots collected from the imaging dishes at each time point (0 min, 5 min, 10 min, and 24 h) during macrophage interaction experiments.

### Hyperspectral Data Analysis

The hypercubes for each sample (786 × 690 × 30) were analyzed in a Python environment. The reflectance of the data set was calculated using the formula given by eq 1 where *R* are the corrected reflectance values of the sample (ranging from 0 to 1) for each spatial pixel (1 × 1 × 30), *I*_raw_ are the raw reflectance intensity values for the sample directly imaged by the system, *I*_dark_ are the reflectance intensity values of a dark background under no illumination. Similarly, the absorbance values, *A*, are calculated as given by eq 2.

1 $$R=\left(\frac{I_{\mathrm{raw}}-I_{\mathrm{dark}}}{I_{\mathrm{white}}-I_{\mathrm{dark}}}\right)\times 0.99$$

2 $$A=-\log_{10}\left(R\right)$$

The hyperspectral data cube containing both cells and AuNSs was cross-correlated with reference data sets, one containing hyperspectral data for only cells and the other only AuNSs, as defined in eqs 3 and 4, respectively, to isolate and identify the contributions of cellular and AuNS components within the samples containing mixture of the two. Prior to cross-correlation, each sample hypercube was cropped to 400 × 400 × 30 to minimize optical glare and eliminate edge-related artifacts.

3 $$R_{\mathrm{AuNS},\lambda }=\left(R_{\mathrm{AuNS}+\mathrm{cells}}\times R_{\mathrm{AuNS}}\right)\left[\lambda \right]$$

4 $$R_{\mathrm{cells},\lambda }=\left(R_{\mathrm{AuNS}+\mathrm{cells}}\times R_{\mathrm{cells}}\right)\left[\lambda \right]$$

Hyperspectral data cubes were processed in Python (v3.9) using a custom pipeline designed to analyze AuNS spectra in THP-1 cell mixtures. The pipeline comprises three components: cross-correlation, spectral metrics, and UMAP visualization. Cross-correlation was performed with *scipy.signal.correlate* to compare spectra from AuNS-cell mixtures against reference AuNS spectra, yielding normalized correlation coefficients that indicate spectral similarity. For each wavelength slice (λ) of the hyperspectral cube, three metrics were computed using *numpy* and *skimage.measure.shannon*_*entropy*: (1) sum of intensity (*I*_sum_), representing total scattering intensity to reflect LSPR strength and particle concentration; (2) standard deviation (σ), capturing intensity variation to identify aggregation or heterogeneity; (3) entropy (*E*), measuring spectral complexity to detect scattering changes due to cellular interactions; and (4) Bright-pixel area (Area_otsu_): computed using Otsu thresholding to segment high-intensity regions, representing the spatial fraction of bright scattering domains associated with aggregated or densely clustered AuNS. The physical interpretation and stability relevance of each metric are summarized in Table 1. These metrics were aggregated into a feature matrix, reduced to 2D projections using *umap-learn* (configured with *n*_*neighbors* = 15, *min*_*dist* = 0.1) to cluster AuNS by formulation and stability. The silhouette coefficient for each UMAP embedding was computed using *sklearn.metrics.silhouette*_*score*, providing a quantitative measure of cluster separation ranging from −1 (poor separation) to +1 (perfect separation); values above 0.5 are interpreted as moderate-to-strong cluster structure.

**Table 1:** Definition and Physical Interpretation of Hyperspectral Imaging Metrics

metric	definition	physical meaning	stability interpretation
summed intensity, *I*_sum_	∑_*i*∈mask_b__ *I*_*i*,b_ per band	total plasmonic scattering cross-section; elevated by high particle concentration or coupling-enhanced extinction	declining: internalization or plasmon red-shift beyond detection window. stable: surface-accessible population preserved
standard deviation, σ	$\sqrt{\frac{1}{N_{b}}\sum_{i\in {\mathrm{mask}}_{b}}{\left(I_{i,b}-{\mathrm{I̅}}_{b}\right)}^{2}}$ per band	spatial scattering heterogeneity. high: mixed dispersed/aggregated populations. low: uniform field	rising σ: emergence of mixed aggregation states. High *I*_sum_ + low σ = stable dispersed population; low *I*_sum_ + low σ = uniform aggregation or clearance
Shannon entropy, *E*	–∑_*i*_ *p*_*i*,b_ log_2_ *p*_*i*,b_ per band	distributional complexity of pixel intensities; high *E* = particles in multiple coexisting optical states; low *E* = narrow unimodal distribution	decreasing *E*: population converging to one dominant optical state via completed aggregation or clearance of heterogeneous subpopulations
bright-pixel area, Area_otsu_	∑_*x*,*y*_ **1**[*I*_b_(*x*, *y*) > *T*_otsu,b_] per band	spatial footprint of plasmonic scatterers; reflects particle loading density and spatial distribution relative to intracellular compartmentalization	contracting area over time: internalization and perinuclear lysosomal sequestration

## Results

Prior to macrophage exposure experiments, all four formulations of Si-AuNS (N1–N4) were characterized by TEM, DLS, PDI, ζ-potential and UV–vis to confirm successful synthesis and colloidal stability. TEM images showed that nanostars synthesized with increasing seed volumes were progressively smaller and exhibited less pronounced tip structures (Figure 1b). Hydrodynamic diameters measured by DLS decreased monotonically with increasing seed volume, from 126.1 ± 25.5 nm for N1 (0 mL) to 63.4 ± 0.8 nm for N4 (6 mL), while TEM-measured diameters followed the same trend (100.5, 83.7, 71.9, and 62.6 nm for N1–N4, respectively) (Figure 1c). The DLS–TEM gap decreased from 25.5 nm at 0 mL to 0.8 nm at 6 mL seed volume (Figure 1d), indicating increasingly compact, spheroid-like morphology at higher seed volumes. Bare AuNS and Si-AuNS showed comparable hydrodynamic diameters at 2, 4, and 6 mL seed volumes, confirming that silica coating did not substantially alter particle size (Figure S1). All formulations exhibited PDI values below 0.2 (range: 0.153–0.168) (Figure S3), and Si-AuNS (−15.3 to −32.3 mV) had consistently more negative ζ-potentials than bare AuNS (−15.3 to −25.3 mV) (Figure S4).

**Figure 1:**
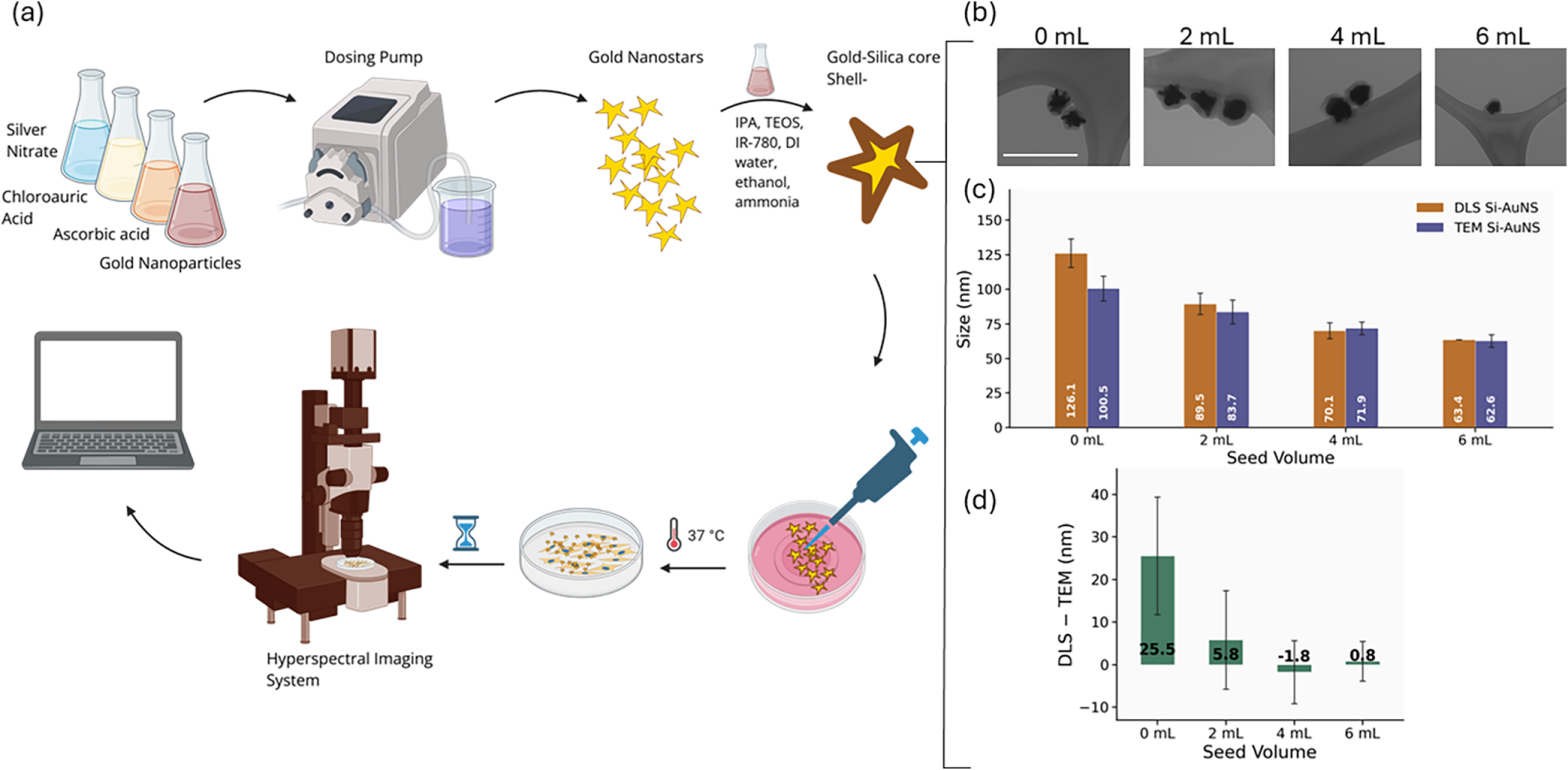
(a) Schematic workflow for the synthesis, functionalization, and analysis of AuNSs in immune-cell microenvironments using hyperspectral sensing; (b) representative TEM images of Si-AuNS synthesized with increasing seed volumes (0, 2, 4, and 6 mL); scale bar = 200 nm; (c) hydrodynamic diameter measured by DLS (amber) and physical diameter measured by TEM (blue) for Si-AuNS across seed volumes (mean ± SD); (d) DLS–TEM gap decreased with increasing seed volume indicating improved colloidal stability and more uniform silica shell formation for higher seed volume particles.

Colloidal stability of Si-AuNS was assessed over 24 h in deionized water, PBS, and RPMI-1640 supplemented with 10% FBS (Figure S2). For N2–N4, the hydrodynamic diameters varied by less than 15 nm across all time points (0 h, 0.5 h, 24 h) in all three media (Figure S2b­(ii–iv)). For N1, the hydrodynamic diameter in water decreased from 171.4 nm at 0 h to 126.1 nm at 0.5 h before stabilizing. The hydrodynamic diameter of N1 in PBS and RPMI+FBS remained consistent over time at ∼120–132 nm (Figure S2b­(i)). PDI values remained below 0.2 throughout (Figure S3), and ζ-potential values ranged from −25.1 to −33.1 mV with no systematic trend (Figure S4), confirming that all four formulations were colloidally stable prior to cell exposure.

The four AuNS formulations were examined through hyperspectral reflectance imaging, UV–vis spectroscopy, DLS, and ζ-potential measurements during their interaction with M0 macrophages at four time points (0 min, 5 min, 10 min, and 24 h). Wide-field hyperspectral reflectance images at 600 nm revealed temporal and formulation-dependent changes in scattering intensity across the cell monolayer (Figure 2). N1 and N2 showed bright, clustered scattering regions at early time points that became increasingly diffuse and heterogeneous over time; most notably, N2 showed a bright, localized high-reflectance cluster at 24 h. N3 and N4 displayed more spatially uniform reflectance distributions throughout, with N4 showing the least overall change. Reflectance decreased progressively from 0 min to 24 h for all formulations, with the most pronounced attenuation observed for N2 and N3 (Figures 2; S12–S19a,c).

**Figure 2:**
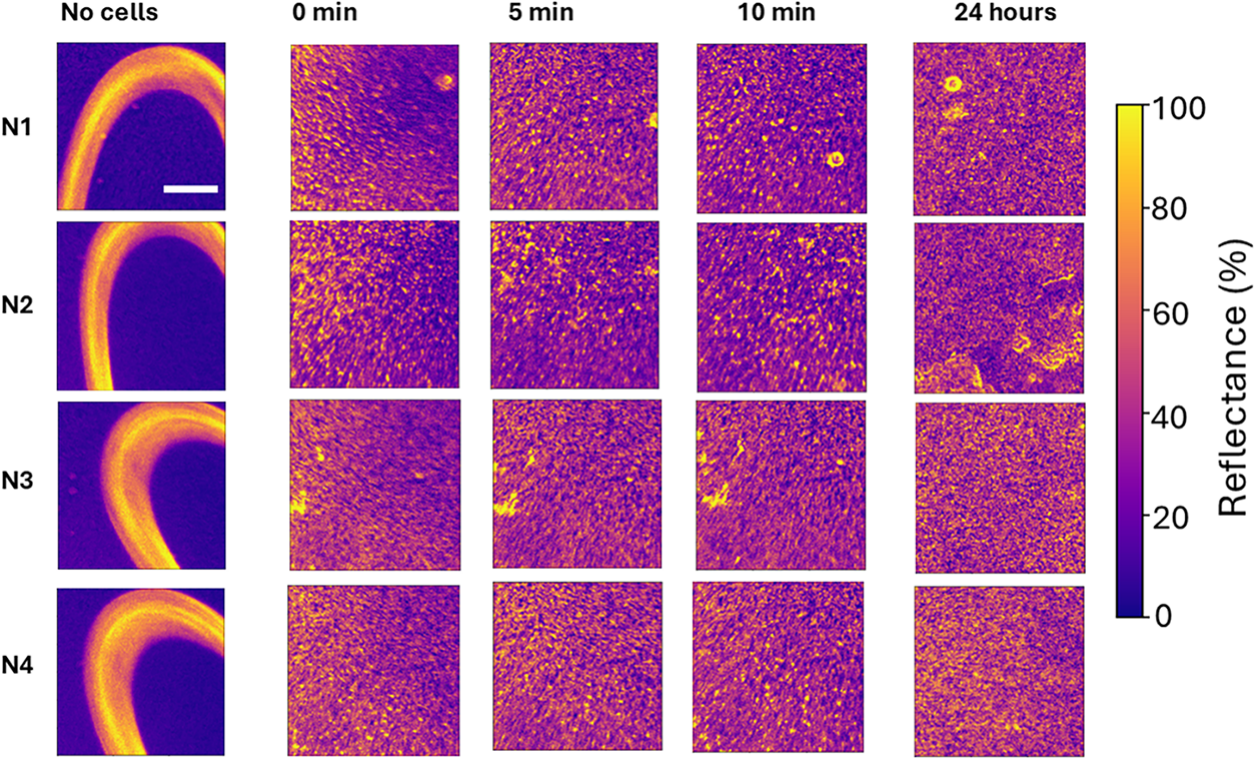
Normalized reflectance images (calculated using eq 1) showing control AuNS samples (column 1) and time-dependent interactions between AuNS and M0 macrophages at 600 nm. The images in column 1 show reflectance from AuNS solutions with glare caused by surface reflections. The glare regions are cropped for analysis. Scale bar = 1 mm.

Reflectance spectra extracted from the hyperspectral data showed a decrease in reflectance in the presence of cells compared to cell-free controls for all formulations (Figure 3a–d). After 0 min exposure, N1, N3, and N4 each showed a ∼5–10% reflectance reduction relative to cell-free controls at red wavelengths (≥600 nm); for N1, this reduction extended to shorter wavelengths as well (Figure 3e). For N2, reflectance values were comparable to the cell-free control below 580 nm but diverged above 600 nm (Figure 3b). Spectral attenuation at 600–700 nm became progressively more pronounced across all groups at 24 h (Figure 3a–d). Reflectance heatmaps quantified this decline across 500, 600, and 700 nm: at 500 nm, N1 decreased from 33.5 to 21.3%, N2 from 26.4 to 19.3%, and N3 from 27.7 to 19.5% (Figure 3e–g).

**Figure 3:**
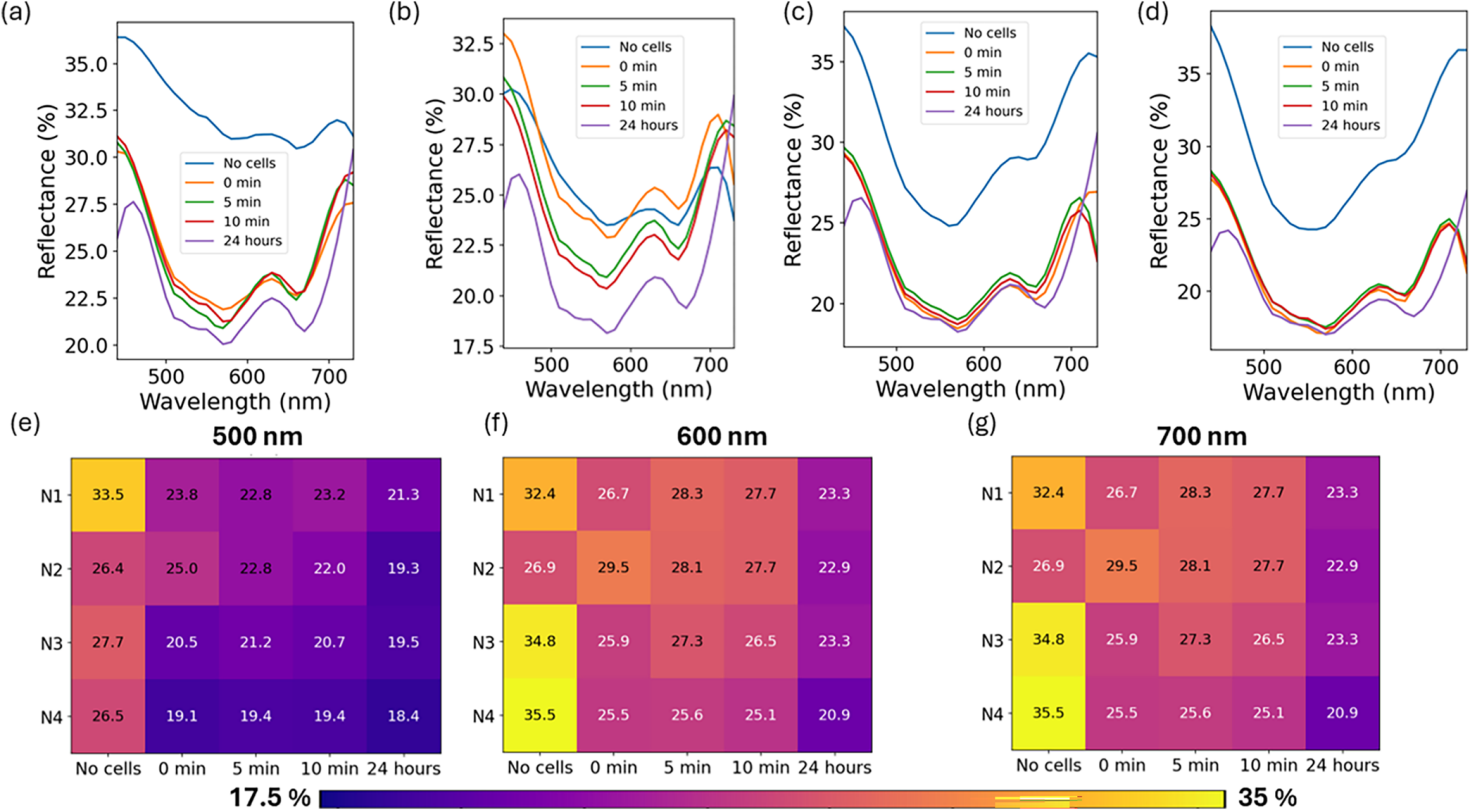
Reflectance spectra at different interaction time points for cell free and cell-interacted (a) AuNS N1, (b) AuNS N2, (c) AuNS N3, and (d) AuNS N4. (e) Heatmap of reflectance values at three representative wavelengths bands of 500 nm, 600, and 700 nm for AuNS N1, (f) AuNS N2, and (g) AuNS N3 to compare AuNS without cells and at 0 min, 5 min, 10 min, and 24 h of culture of cells with AuNS (columns).

Apparent absorbance spectra from hyperspectral imaging showed a consistent, time-dependent increase in absorbance across all formulations (Figure 4a–d). The most pronounced OD changes occurred after 24 h. N1 and N4 showed increases of approximately 0.06–0.10 OD units relative to cell-free controls in the 600–700 nm window. The spectral shift toward red wavelengths at 24 h was most pronounced for N2 and N3. Absorbance heatmaps confirmed this: at 600 nm, N1 increased from 0.62 to 0.70 (Δ = 0.08), N2 from 0.72 to 0.74 (Δ = 0.02), and N3 from 0.66 to 0.74 (Δ = 0.08) over the full experimental period (Figure 4e–g).

**Figure 4:**
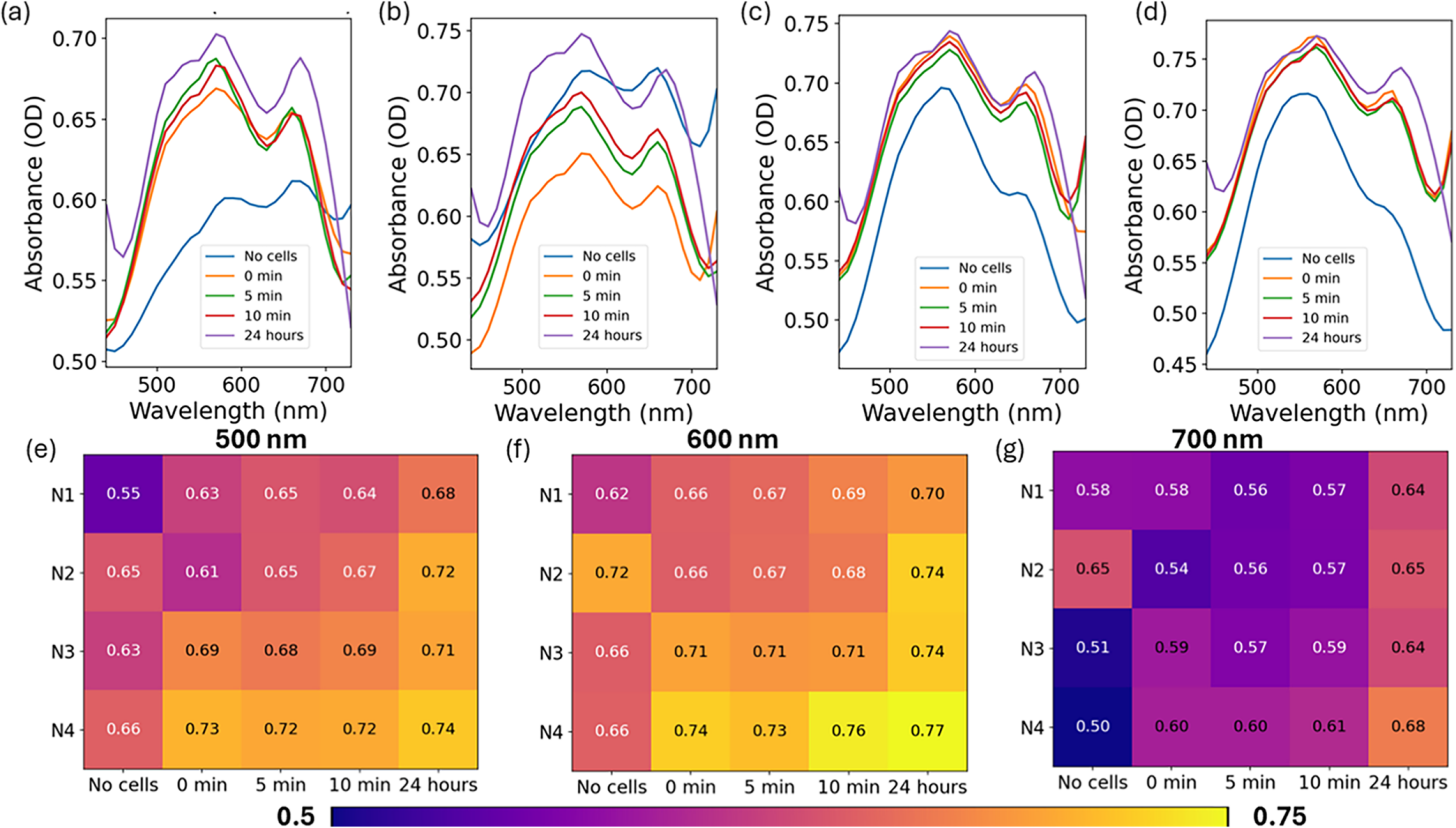
Apparent absorbance spectra at different interaction time points for cell free and cell-interacted (a) AuNS N1, (b) AuNS N2, (c) AuNS N3, and (d) AuNS N4. (e) Heatmap of absorbance values at three representative wavelengths bands of 500 nm, 600 nm, and 700 nm for AuNS N1, (f) AuNS N2, and (g) AuNS N3 to compare AuNS without cells and at 0 min, 5 min, 10 min, and 24 h of culture of cells with AuNS (columns).

UV–vis characterization of bare AuNS and Si-AuNS prior to cell exposure confirmed seed-volume-dependent SPR peaks of 970, 630, 545, and 540 nm for Si-AuNS N1–N4, respectively, with silica coating producing a modest red-shift relative to bare AuNS for all formulations (Figure 5a­(i–iv)). UV–vis spectra of Si-AuNS recovered from macrophage-interacted dishes revealed time-dependent spectral changes that differed markedly across formulations (Figure 5b). N3 exhibited the largest absolute red-shift and greatest peak broadening at 24 h (Figure 5b­(iii)). N2 showed moderate peak broadening most apparent at 24 h (Figure 5b­(ii)). N1 showed broadening and peak flattening by 24 h that was spectrally uniform across all wavelengths, distinct from the NIR-selective broadening of N2 and N3 (Figure 5b­(i)). N4 retained the narrowest peak and smallest spectral shift at all time points (Figure 5b­(iv)).

**Figure 5:**
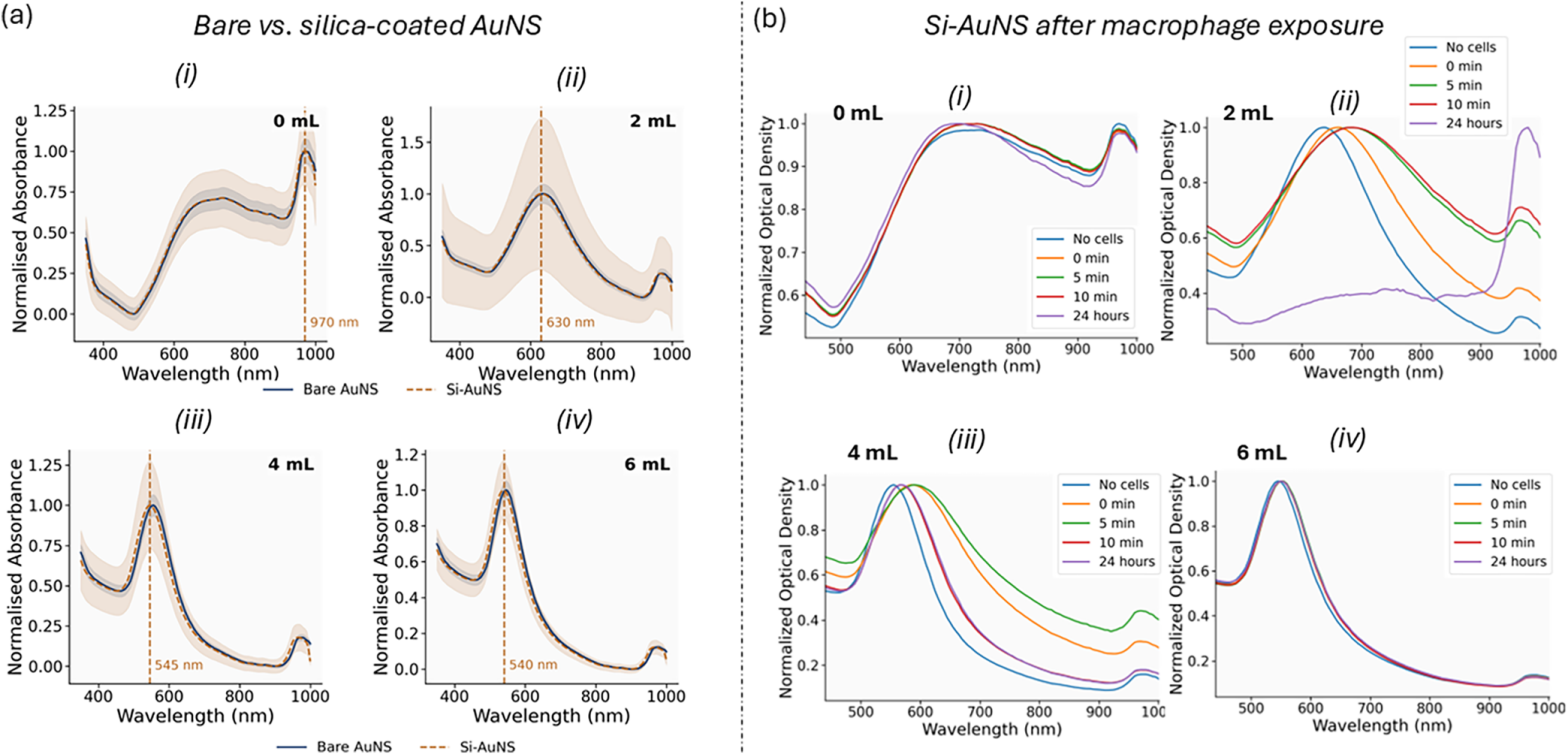
(a) Normalized absorbance spectra of bare AuNS (solid blue) and silica-coated AuNS (Si-AuNS, dashed orange) for all four seed volume formulations (prior to interaction with the cells): (i) 0 mL (N1), (ii) 2 mL (N2), (iii) 4 mL (N3), and (iv) 6 mL (N4). Shaded regions indicate ± standard deviation across replicates. Annotated dotted lines indicate the SPR peak wavelength of each Si-AuNS formulation (970, 630, 545, and 540 nm for N1–N4 respectively). (b) Normalized absorbance spectra of Si-AuNS recovered from macrophage culture dishes at five conditions: no-cell control, 0 min, 5 min, 10 min, and 24 h postexposure, for N1 (0 mL), N2 (2 mL), N3 (4 mL), and N4 (6 mL).

UMAP analysis of hyperspectral reflectance data revealed formulation- and time-dependent divergence among AuNS. Four spatial–spectral features—*I*_sum_(*x*,*y*), σ­(*x*,*y*), *E*(*x*,*y*), and *Area*(*x*,*y*)_otsu_—were extracted from cross-correlated AuNS-specific image stacks (Figure 6a) and projected to two dimensions. At 0 min, four well-separated, formulation-specific clusters confirmed distinct optical fingerprints prior to cell exposure (Figure 6b­(i)). At 5 min, partial overlap between N1 and N3 clusters emerged while N2 and N4 remained compact (Figure 6b­(ii)). By 10 min, N1 and N3 overlap broadened further while N4 remained well-separated (Figure 6b­(iii)). At 24 h, N1 and N3 exhibited the greatest internal dispersion and cluster drift, N2 showed partial recovery toward a more compact distribution, and N4 maintained a tight, localized cluster (Figure 6b­(iv)). The global spectral spread peaked at intermediate time points before contracting at 24 h, and the silhouette coefficient rose from 0.670 at 0 min to a peak of 0.879 at 10 min before settling at 0.730 at 24 h (Figure 6d). UMAP projections for immune cells showed diffuse, fully overlapping distributions at all time points with no formulation-specific structure (Figure 6c), and silhouette coefficients for cell embeddings ranged from 0.336 to 0.357 with no temporal trend (Figure S9b), in contrast to nanostar embeddings which exceeded 0.5 throughout and peaked at 0.879 at 10 min (Figure S9a).

**Figure 6:**
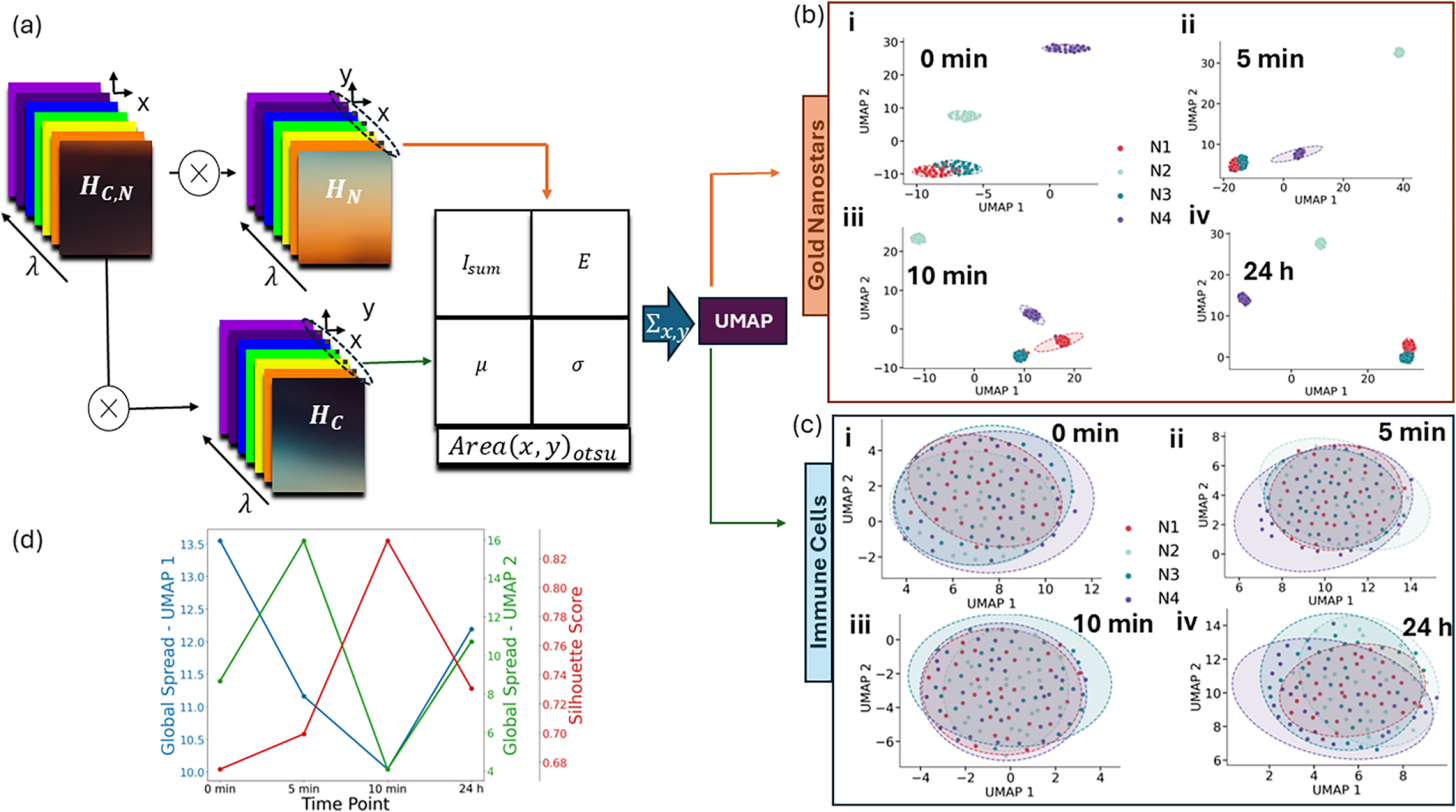
Cross-correlation workflow and UMAP-based hyperspectral analysis of AuNS and immune-cell interactions. (a) Schematic representation of hyperspectral data cubes: *H*_CN_(mixed AuNS + cell sample), *H*_N_ (AuNS-only reference), and *H*_C_ (cell-only reference). Cross-correlation of these data sets isolates the nanoparticle-specific and cellular spectral components. (b) UMAP projections of AuNS-only cross-correlated hyperspectral data (*R*_AuNS,λ_) at (i) 0 min, (ii) 5 min, (iii) 10 min, and (iv) 24 h for four AuNS formulations (N1–N4). (c) UMAP projections of immune-cell-only cross-correlated hyperspectral data (*R*_cells,λ_) at (i) 0 min, (ii) 5 min, (iii) 10 min, and (iv) 24 h for four AuNS formulations (N1–N4). N1 (amaranth red, #d72631), N2 (mint green, #a2d5c6), N3 (teal blue, #077b8a), and N4 (royal purple, #5c3c92), correspond to seed volumes of 0, 2, 4, and 6 mL. (d) UMAP metrics showing temporal evolution of the global spectral spread and silhouette coefficient across formulations for AuNS-only cross-correlated hyperspectral data.

ζ-potential measurements following macrophage incubation showed formulation-dependent surface charge evolution (Figure 7a). All formulations initially exhibited moderately negative surface charges (N1: −26.4 ± 3.12 mV; N2: −30.63 ± 1.16 mV; N3: −29.5 ± 0.2 mV; N4: −30.07 ± 0.29 mV). N1 and N3 showed transient increases toward less negative values followed by sharp decreases in magnitude over time (Figure 7a­(i),(iii)), while N2 and N4 retained consistently negative values throughout (Figure 7a­(ii),(iv)). Hydrodynamic size measurements revealed distinct aggregation profiles for each formulation (Figure 7b). N2 showed the largest absolute size increase, rising from 81.18 ± 8.86 nm at 0 min to ∼1200 nm at 5 min before partially recovering to ∼950 nm at 10 min (Figure 7b­(ii)). N3 exceeded 1400 nm by 10 min with less recovery (Figure 7b­(iii)). N1 fluctuated modestly between ∼150 and 190 nm throughout (Figure 7b­(i)). N4 remained below 160 nm at all time points, reaching 73.44 ± 5.89 nm at 24 h (Figure 7b­(iv)).

**Figure 7:**
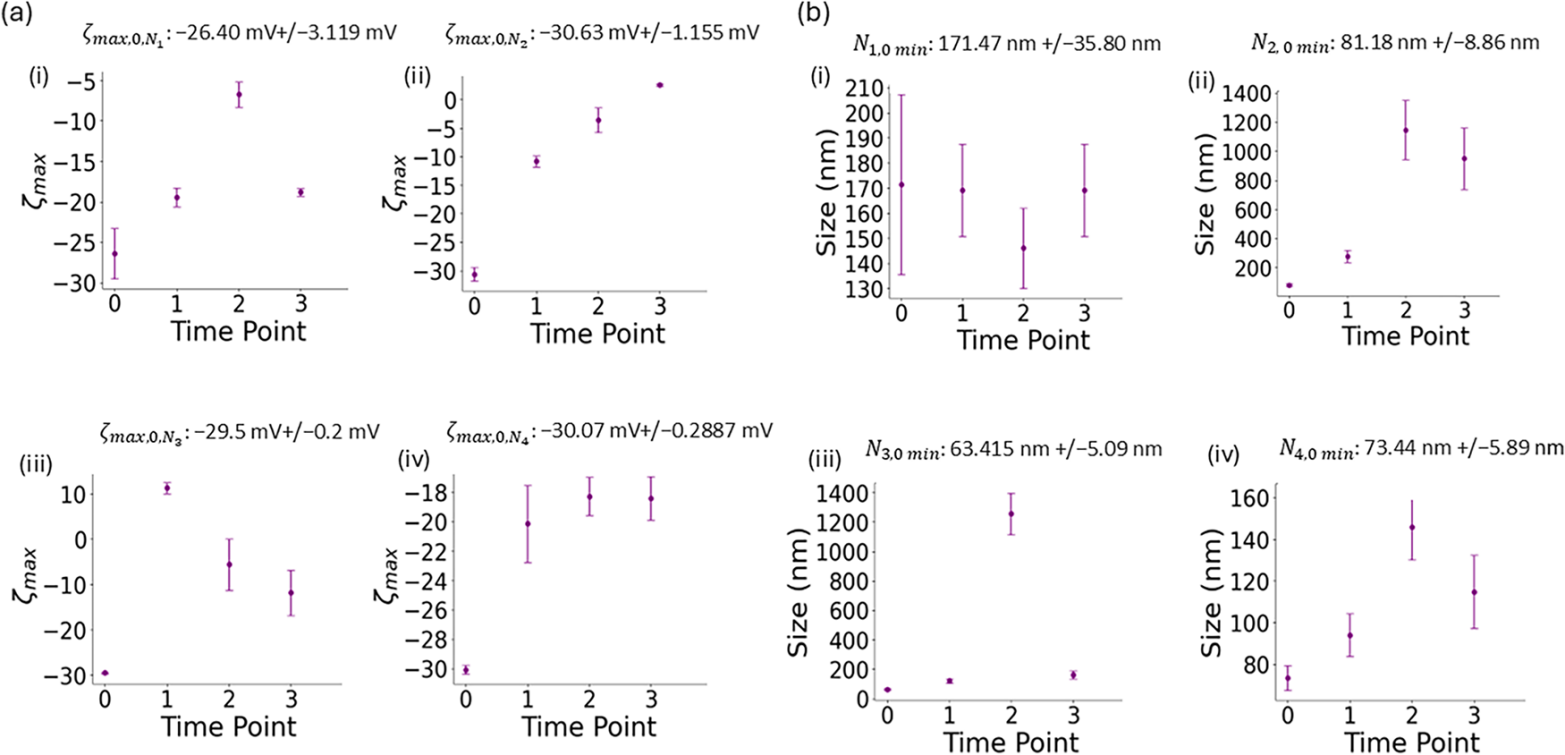
ζ-potential and hydrodynamic diameter of four AuNS formulations (N1–N4) following incubation with immune cells for varying durations 0 min, 5 min, 10 min, and 24 h (time points 0, 1, 2, and 3 respectively), measured after nanoparticle recovery from the cellular environment. (a)­(i–iv) ζ-potential (ζ_*max*_) of AuNS N1–N4, respectively, showing formulation-dependent surface charge alterations over time. N1 and N3 exhibit a marked reduction in ζ-potential magnitude, indicating destabilization, while N2 and N4 retain relatively stable surface charges. (b)­(i–iv) Hydrodynamic diameter profiles of N1–N4, respectively, determined by dynamic light scattering (DLS). N3 undergoes significant aggregation, with particle size exceeding 1400 nm by 10 min, while N2 and N4 maintain more consistent sizes over the incubation period, suggesting enhanced colloidal stability.

Wavelength-resolved spectral metrics across 30 hyperspectral channels (440–730 nm) provided per-band optical evolution for all four formulations (Figures S12, S14, S16, S18 for nanostar stacks; Figures S13, S15, S17, S19 for cell stacks). *I*_sum_ declined progressively over 24 h across all formulations (Figure S5a). σ followed a nonmonotonic trajectory for N1, N2, and N3, decreasing from 0 to 10 min and increasing at 24 h, while N4 remained consistently the lowest (Figure S5b). Entropy and *Area*_otsu_ declined at 24 h for all formulations, with N4 showing the steepest contraction in bright-pixel area from ∼4000 to ∼3600 px (Figures S5c,d). *Z*-scored heatmaps showed N2 had the highest *I*_sum_ *z*-score (∼ +2) and N4 at the lowest (∼ −2) (Figure S7), while cell-derived stacks showed no formulation-dependent patterns (Figure S8). Centroid displacement analysis (Figure S10) showed N2 had the largest cumulative path length (121.59 au) driven by a coherent spike to ∼52 au at 5 min, while N1 (65.18 au) and N3 (63.40 au) showed progressive drift with greater within-group spread (Figure S11), and N4 showed the smallest displacement (55.02 au) and tightest cluster throughout.

The key findings from the hyperspectral pipeline were independently corroborated across all physicochemical modalities. N4 was the most stable by every measure: most negative retained ζ-potential (−30.07 ± 0.29 mV at baseline, minimal change at 24 h), smallest hydrodynamic size at 24 h (73.44 ± 5.89 nm), narrowest UV–vis peak, and smallest UMAP displacement. N3 showed the most severe instability: DLS size exceeding 1400 nm at 10 min (Figure 7b­(iii)) and the largest UV–vis red-shift and peak broadening at 24 h (Figure 5b­(iii)). N1 and N2 exhibited distinct intermediate behaviors: N2 underwent acute, large-magnitude aggregation by DLS (reaching ∼1200 nm at 5 min) but showed the smallest OD change at 600 nm (Δ = 0.02) and a coherent, recoverable UMAP shift; N1 remained small by DLS (≤190 nm) but showed progressive ζ-potential destabilization, broader spectral changes at short wavelengths, and greater within-group optical heterogeneity in UMAP space.

## Discussion

The central finding of this work is that seed volume, and correlated nanostar morphology, is a primary determinant of AuNS colloidal and optical stability in macrophage microenvironments. Increasing seed volume produced progressively smaller, more compact nanostars with less prominent tips, as captured by the convergence of the DLS–TEM gap from 25.5 nm to 0.8 nm (Figure 1b,d). Across all modalities, N4, the most compact formulation, was the most stable across every modality. N3, despite having a smaller baseline hydrodynamic diameter than N1 (63 nm vs 171 nm), was the most severely destabilized, underlining that compactness and tip geometry govern stability in the cellular microenvironment more than absolute particle size.^14,25^ N1 and N2 displayed distinct instability profiles: N1 underwent gradual, progressive destabilization across all time points, while N2 underwent acute early aggregation followed by partial recovery.

The time points were selected to capture distinct and biologically meaningful phases of nanoparticle-cell interaction. The 0 min images reflect a postincubation baseline following the 30 min AuNS exposure and PBS wash, at which point protein corona formation has already initiated. Protein corona assembly on gold nanoparticles occurs within seconds to minutes of exposure to serum-containing media, with an evolution from a loosely attached toward an irreversibly bound corona documented by UV–vis, DLS, and ζ-potential measurements under in vitro cell culture conditions.^26–28^ The 5 min point was chosen to capture the earliest optically detectable consequences of corona maturation and surface-mediated aggregation. The Vroman effect, whereby initially adsorbed proteins are progressively exchanged for higher affinity proteins, is expected to produce measurable changes in the dielectric environment around the nanoparticle surface on a time scale of seconds to minutes.^26,27^ The 10 min point was included to determine whether the optical state at 5 min represents a transient peak or a sustained trajectory, and to capture the onset of active cellular association, as gold nanoparticles have been shown to bind to macrophage cell surfaces within minutes of exposure.^29,30^ The 24 h time point represents the late-stage optical outcome after prolonged exposure, providing a biologically meaningful end point that contrasts with the acute response captured at earlier time points.

The wide-field reflectance images (Figure 2) provide macroscale spatial context that ensemble measurements cannot offer. The bright, localized cluster visible for N2 at 24 h is a direct spatial manifestation of large-scale agglomeration confirmed by DLS (Figure 7b­(ii)), and its persistence as a discrete high-reflectance domain suggests that a subpopulation of aggregated particles remained surface-associated rather than fully internalized. The progressive loss of reflectance uniformity for N1 and N3 is consistent with their ζ-potential destabilization (Figure 7a­(i), (iii)), while N4’s spatially stable distribution reflects its electrostatic resilience throughout the experiment. The reflectance attenuation at 600–700 nm (Figure 3a–d) and corresponding absorbance increases (Figure 4a–d) are characteristic signatures of LSPR hybridization into collective lower-energy modes upon nanoparticle proximity (<10 nm).^31,32^ The modest ∼5–10% reflectance reduction at 0 min most likely reflects membrane adsorption and early corona formation altering the local dielectric environment rather than interparticle coupling,^30,33,34^ while the pronounced red-shifts at 24 h are consistent with progressive clustering in acidic endocytic compartments.^31,35,36^ The reflectance and absorbance heatmaps (Figures 3e–g, 4e–g) enable quantification of the magnitude and time-dependence of these changes across all formulations and wavelengths in a single visualization. These spectral changes are independently consistent with the precell stability characterization, which confirmed that all Si-AuNS formulations maintained stable hydrodynamic diameters, PDI < 0.2, and consistently negative ζ-potentials in DI water, PBS, and RPMI+10% FBS over 24 h prior to cell exposure (SI Figures S2–S4), ruling out inherent colloidal instability as a contributing factor.

The seed-volume-dependent SPR peaks at 970, 630, 545, and 540 nm for N1–N4 (Figure 5a­(i–iv)), reflect the influence of tip geometry on plasmonic response. The far-red SPR of N1 at 970 nm arises from electromagnetic field enhancement at its longer, sharper tips, while the blue-shifted peaks of N3 and N4 are consistent with a more spheroid-like morphology.^14^ These distinct fingerprints are the physical basis for the well-separated N1–N4 clusters in UMAP space at 0 min (Figure 6b­(i)). By 24 h, the spectral changes in UV–vis spectra (Figure 5b) of recovered Si-AuNS reveal mechanistically distinct instability pathways for N1 and N2. N3′s extreme red-shift and NIR broadening (Figure 5b­(iii)) reflect the most extensive interparticle coupling, consistent with DLS sizes exceeding 1400 nm (Figure 7b­(iii)). N2’s NIR-selective broadening followed by partial spectral recovery (Figure 5b­(ii)) mirrors its DLS profile of rapid agglomeration to ∼1200 nm followed by partial size reduction (Figure 7b­(ii)), suggesting sedimentation of the largest aggregates. By contrast, N1 shows spectrally flat attenuation across all wavelengths rather than NIR-selective coupling signatures (Figures 5b­(i); S12 and S13), indicating that particle number density reduction through clearance or internalization dominates over coupling-induced spectral reshaping consistent with N1’s relatively stable DLS size (≤190 nm) despite its progressive ζ-potential loss. This broadband attenuation, distinct from the NIR-selective coupling signatures of N2 and N3, is further consistent with N1’s larger DLS–TEM gap (25.5 nm vs 0.8 nm for N4), which implies a thicker silica shell with greater sensitivity to refractive-index perturbation by protein corona formation at the nanoparticle surface. N4’s minimal spectral evolution reflects suppression of both pathways by its strong electrostatic stability (Figure 7a­(iv),b­(iv)).

These data reveal two distinct instability mechanisms among the intermediates. N2 undergoes acute, coherent aggregation: it generates the largest absolute DLS size increase and the largest UMAP centroid displacement (121.59 au, Figure S10) but maintains a tight, low-spread cluster throughout (Figure S11), indicating a population-wide, uniform optical shift rather than heterogeneous divergence. N1, despite remaining small by DLS, undergoes gradual, progressive surface charge loss (Figure 7a­(i)) and produces greater within-group optical heterogeneity in UMAP space (Figure S11), consistent with a slower but more spatially diverse destabilization process. These two failure modes, acute aggregation versus gradual surface degradation, would be indistinguishable by ensemble UV–vis or DLS alone but are clearly resolved by the hyperspectral UMAP framework. N3, though starting with a smaller baseline diameter than N1, combines both failure modes: rapid charge loss (Figure 7a­(iii)) and the most extreme aggregation (Figure 7b­(iii)), which together produce the most severe and irreversible optical transformation across all modalities. The trade-off between these stability regimes has direct relevance for biomedical applications: high colloidal stability (N4) ensures reproducible spectral signatures for imaging and diagnostics, while controlled or acute aggregation may transiently enhance local electromagnetic fields for photothermal therapy applications. We note that this framework complements rather than replaces conventional physicochemical metrics such as DLS or ζ-potential; each modality provides information inaccessible to the other. Establishing formal quantitative correlations between imaging-derived metrics and physicochemical parameters would require additional biological replicates and is identified as a direction for future work.

The UMAP framework captures the full temporal trajectory of these optical changes in a single, interpretable representation (Figure 6b). The nonmonotonic global spread peaking at 5 min and contracting at 24 h (Figure 6d) reflects maximum interformulation optical diversity during early aggregation kinetics, followed by convergence as unstable formulations approach completed aggregation states. The silhouette coefficient quantifies cluster separation on a scale from −1 (misclassified) to +1 (perfectly separated), with values above 0.5 indicating moderate-to-strong cluster structure. The silhouette coefficient peak of 0.879 at 10 min (Figure S9a) identifies this time point as providing the highest formulation classification accuracy, with practical implications for rapid stability screening. The full trajectory, rising from 0.670 at 0 min to 0.879 at 10 min, then settling at 0.730 at 24 h, reflects initial optical distinctness, maximum interformulation divergence during early aggregation kinetics, and partial reconvergence as unstable formulations approach completed aggregation states, respectively. The formulation-independent, diffuse cell UMAP distributions (Figures 6c; S9b) and near-identical cell-derived metric trajectories (Figures S6 and S8) confirm that all spectral drift in the nanostar embeddings originates from AuNS-specific optical transformations rather than variable cellular responses,^15,37,38^ validating the cross-correlation-based spectral separation approach. It is worth noting that the optical contrast in this framework is dominated by AuNS plasmonic scattering; the platform therefore characterizes nanoparticle optical state primarily, with cellular influences (corona formation, endocytic uptake, lysosomal processing) inferred indirectly from the temporal evolution of spectral signatures rather than imaged directly. The diffuse, fully overlapping cell UMAP distributions (Figure 6c); silhouette scores 0.336–0.357, Figure S9b and the absence of formulation-dependent structure in cell-derived metric heatmaps (Figure S8) confirm that macrophage regions do not contribute AuNS-specific contrast.

Taken together, this work establishes wide-field hyperspectral reflectance imaging with cross-correlation-based spectral unmixing and unsupervised learning as a reliable, label-free platform for resolving formulation-dependent nanoparticle stability and distinct instability mechanisms in immune-cell microenvironments, capabilities that go beyond what conventional ensemble spectroscopy can provide. We note that this framework complements rather than replaces conventional physicochemical metrics such as DLS or ζ-potential; each modality provides information inaccessible to the other, and formal quantitative correlation between imaging-derived metrics and physicochemical parameters remains a direction for future work. Future experiments employing intracellular TEM or correlative confocal/electron microscopy on nanoparticle-exposed cells would also directly confirm the endosomal clustering mechanism inferred here from spectral and physicochemical data.

## Conclusions

This study demonstrates that wide-field hyperspectral reflectance imaging, combined with cross-correlation-based spectral unmixing and UMAP dimensionality reduction, provides a scalable, label-free platform for resolving the formulation-dependent AuNS stability in immune-cell microenvironments. Across four Si-AuNS formulations, seed volume, through its correlation with nanostar morphology and tip geometry, emerged as the primary determinant of colloidal and optical stability: the most compact formulation (N4) was the most stable across all modalities, while N3 underwent the most severe aggregation despite its smaller baseline diameter, underscoring that compactness governs stability more than absolute particle size alone. N1 and N2 exhibited mechanistically distinct instability profiles; N1 underwent gradual progressive surface degradation, while N2 showed acute coherent aggregation followed by partial recovery, differences that were clearly resolved by the hyperspectral UMAP framework but were indistinguishable by ensemble UV–vis or DLS measurements alone. Four complementary spectral metrics extracted from hyperspectral image stacks captured these differences quantitatively, with independent corroboration from ζ-potential, DLS, and UV–vis spectroscopy confirming the validity of the imaging-based approach. These findings establish design principles linking nanostar geometry and surface chemistry to stability in macrophage microenvironments with direct implications for the rational development of AuNS formulations for immunotherapeutic and diagnostic applications.

## Supplementary Material



## Notes

Size characterization of bare and silica-coated AuNS by DLS and TEM (Figure S1); hydrodynamic size stability of Si-AuNS in physiological media before cell exposure (Figure S2); polydispersity index of bare and silica-coated AuNS before and during stability testing (Figure S3); ζ-potential of bare and silica-coated AuNS before and during stability testing (Figure S4); temporal evolution of hyperspectral feature metrics for AuNS-only cross-correlated image stacks, N1–N4 (Figure S5); temporal evolution of hyperspectral feature metrics for cell-only cross-correlated image stacks, N1–N4 (Figure S6); *z*-scored spectral metric heatmap for AuNS-only cross-correlated image stacks (Figure S7); *z*-scored spectral metric heatmap for cell-only cross-correlated image stacks (Figure S8); UMAP cluster silhouette coefficients for AuNS-only and cell-only cross-correlated image stacks (Figure S9); UMAP centroid displacement analysis for AuNS-only and cell-only cross-correlated image stacks (Figure S10); mean pairwise UMAP cluster distance for AuNS-only and cell-only cross-correlated image stacks (Figure S11); wavelength-resolved hyperspectral metrics for N1 nanostars, AuNS-only cross-correlated data (Figure S12); wavelength-resolved hyperspectral metrics for N1 nanostars, cell cross-correlated data (Figure S13); wavelength-resolved hyperspectral metrics for N2 nanostars, AuNS-only cross-correlated data (Figure S14); wavelength-resolved hyperspectral metrics for N2 nanostars, cell cross-correlated data (Figure S15); wavelength-resolved hyperspectral metrics for N3 nanostars, AuNS-only cross-correlated data (Figure S16); wavelength-resolved hyperspectral metrics for N3 nanostars, cell cross-correlated data (Figure S17); wavelength-resolved hyperspectral metrics for N4 nanostars, AuNS-only cross-correlated data (Figure S18); wavelength-resolved hyperspectral metrics for N4 nanostars, cell cross-correlated data (Figure S19) (PDF)
