# Supplementary material for: Mapping Morphology-Dependent Stability of Gold Nanostars in Immune Cells Using Hyperspectral Imaging
Source: Anal Chem. 2026 Jun 18;98(25):19271–82. doi: 10.1021/acs.analchem.6c03010 (PMC13325451; doi:10.1021/acs.analchem.6c03010)
Supplement: Supplementary file 1 [file ac6c03010_si_001.pdf]

Supporting Information (SI)

Mapping Morphology-Dependent Stability of Gold Nanostars in Immune Cells Using Hyperspectral Imaging

Lakhvir Singh,†,‡ Ngoc Nhu Vu,†,‡ Elizabeth A Bullard,† Erin M Stout,† Samuel Mabbott,† and Alex J Walsh*,†

†Department of Biomedical Engineering, Texas A&M University, College Station, Texas 77843, United States

‡Lakhvir Singh and Ngoc Nhu Vu contributed equally.

Content	Page no.
**Table of Contents**	S1, S2
**Figure S1.** Size characterization of bare and silica-coated AuNS by DLS and TEM.	S2
**Figure S2.** Hydrodynamic size stability of Si-AuNS before cell exposure.	S3
**Figure S3.** Polydispersity index (PDI) of bare and silica-coated AuNS before and during stability testing.	S4
**Figure S4.** Zeta potential of bare and silica-coated AuNS before and during stability testing.	S5
**Figure S5.** Temporal evolution of four hyperspectral feature metrics extracted from cross-correlated AuNS-only image stacks for N1–N4 nanostars interacting with THP-1-derived M0 macrophages.	S6
**Figure S6.** Temporal evolution of hyperspectral feature metrics extracted from cross-correlated cell-only image stacks for THP-1-derived M0 macrophages exposed to N1–N4 nanostar formulations.	S7
**Figure S7.** Heatmap visualization of z-scored spectral metrics for AuNS-only cross-correlated image stacks across four nanostar formulations (N1–N4, rows) and four time points (columns).	S7
**Figure S8.** Heatmap visualization of z-scored spectral metrics extracted from cell-only cross-correlated image stacks.	S8
**Figure S9.** Silhouette coefficient of UMAP cluster embeddings at each time point for (a) AuNS-only and (b) cell-only cross-correlated image stacks.	S8
**Figure S10.** Centroid displacement analysis of UMAP embeddings for (a) AuNS-only and (b) cell-only cross-correlated image stacks.	S9
**Figure S11.** Mean pairwise distance within each formulation's UMAP cluster at each time point for (a) AuNS-only and (b) cell-only cross-correlated image stacks.	S10
**Figure S12**. Wavelength-resolved hyperspectral metrics for N1 nanostars interacting with THP-1-derived M0 macrophages (cross correlated data for the nanostars).	S10
**Figure S13.** Wavelength-resolved hyperspectral metrics for N1 nanostars interacting with THP-1-derived M0 macrophages (cross correlated data for the cells).	S11
**Figure S14.** Wavelength-resolved hyperspectral metrics for N2 nanostars interacting with THP-1-derived M0 macrophages (cross correlated data for the nanostars).	S12

Content	Page no.
**Figure S15.** Wavelength-resolved hyperspectral metrics for N2 nanostars interacting with THP-1-derived M0 macrophages (cross correlated data for the cells).	S13
**Figure S16.** Wavelength-resolved hyperspectral metrics for N3 nanostars interacting with THP-1-derived M0 macrophages (cross correlated data for the nanostars).	S14
**Figure S17.** Wavelength-resolved hyperspectral metrics for N3 nanostars interacting with THP-1-derived M0 macrophages (cross correlated data for the cells).	S15
**Figure S18.** Wavelength-resolved hyperspectral metrics for N4 nanostars interacting with THP-1-derived M0 macrophages (cross correlated data for the nanostars).	S16
**Figure S19**. Wavelength-resolved hyperspectral metrics for N4 nanostars interacting with THP-1-derived M0 macrophages (cross correlated data for the cells).	S17

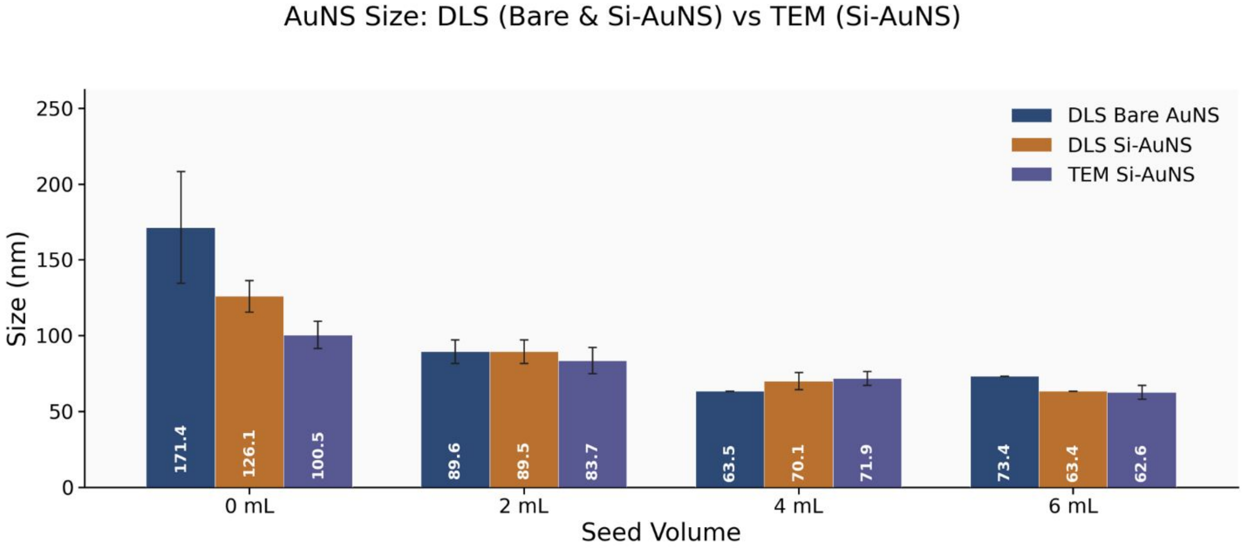

Figure S1. Size characterization of bare and silica-coated AuNS by DLS and TEM. Hydrodynamic diameter measured by DLS for bare AuNS (dark blue) and Si-AuNS (amber), and physical diameter measured by TEM for Si-AuNS (purple), across all four seed volume formulations (0, 2, 4, and 6 mL). Mean values are annotated within each bar; error bars represent ± standard deviation. Particle size decreases with increasing seed volume for all three measurement types. The converging gap between DLS and TEM values at 4-6 mL is consistent with an increasingly compact, spherical morphology and a thinner, more uniform silica shell at higher seed volumes.

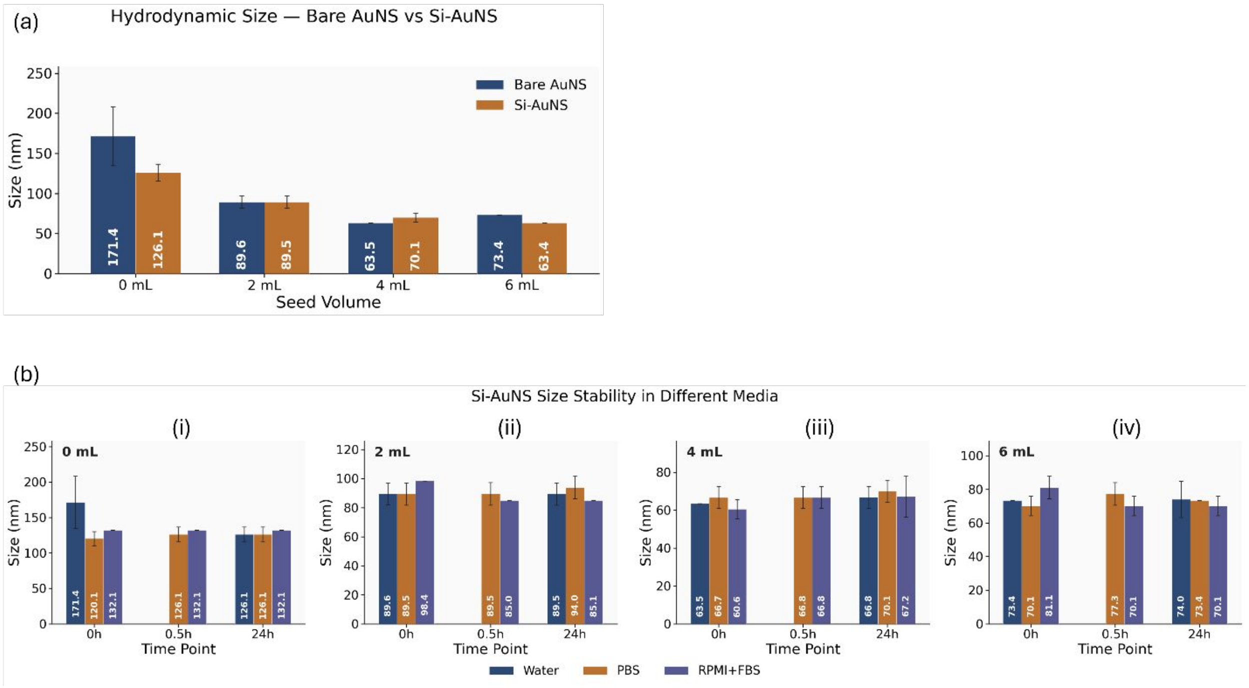

Figure S2. Hydrodynamic size stability of Si-AuNS before cell exposure. (a) Comparison of DLS hydrodynamic diameter for bare AuNS and Si-AuNS across all four seed volume formulations. (b)(i-iv) Hydrodynamic size of Si-AuNS measured in deionized water (dark blue), PBS (amber), and complete RPMI + FBS (purple) at 0 h, 0.5 h, and 24 h for the 0 mL, 2 mL, 4 mL, and 6 mL formulations respectively. Mean values are annotated within each bar; error bars represent ± standard deviation. All formulations maintained stable hydrodynamic sizes across all three media over 24 h, confirming colloidal stability prior to cell exposure and establishing that optical changes observed during cell incubation are not attributable to inherent aggregation in physiological media.

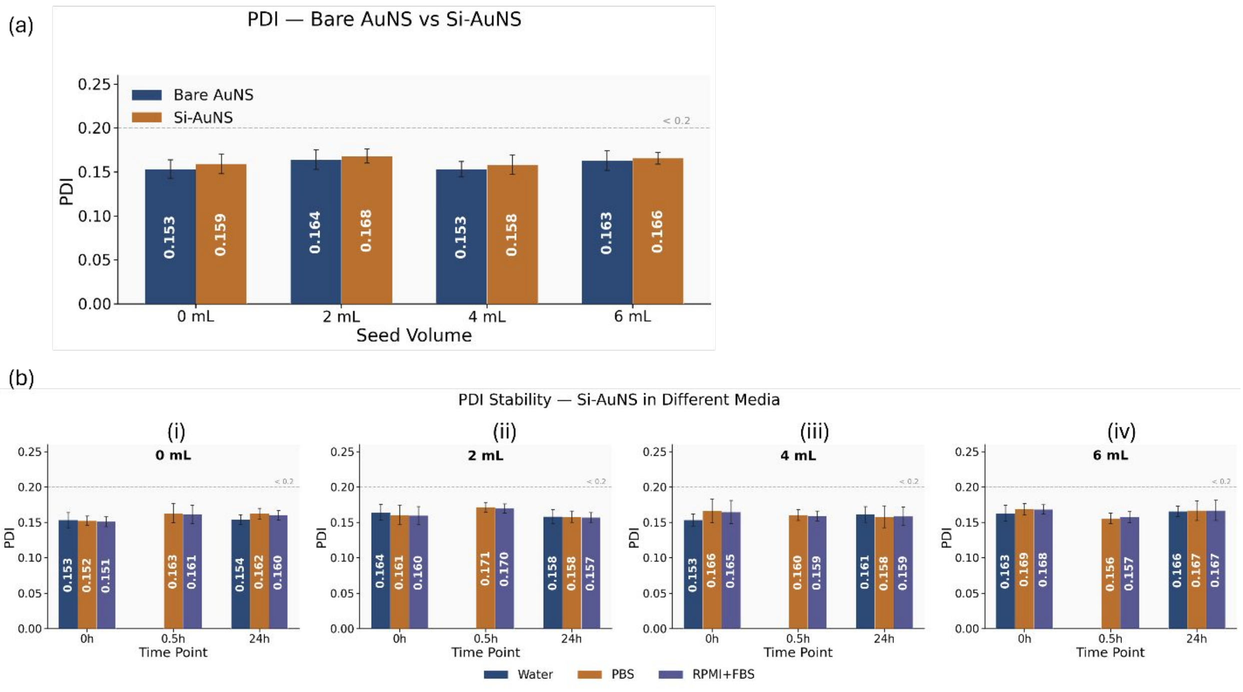

Figure S3. **Polydispersity index (PDI) of bare and silica-coated AuNS before and during stability testing.** (a) PDI values for bare AuNS and Si-AuNS across all four seed volume formulations. The dashed reference line at PDI = 0.2 indicates the threshold below which a distribution is considered monodisperse. All formulations fall below this threshold. (b)(i--iv) PDI stability of Si-AuNS in deionized water (dark blue), PBS (amber), and complete RPMI + FBS (purple) at 0 h, 0.5 h, and 24 h for the 0 mL, 2 mL, 4 mL, and 6 mL formulations respectively. PDI values remained consistently below 0.2 across all media and time points, confirming narrow, monodisperse size distributions and colloidal stability of all Si-AuNS formulations prior to cell exposure.

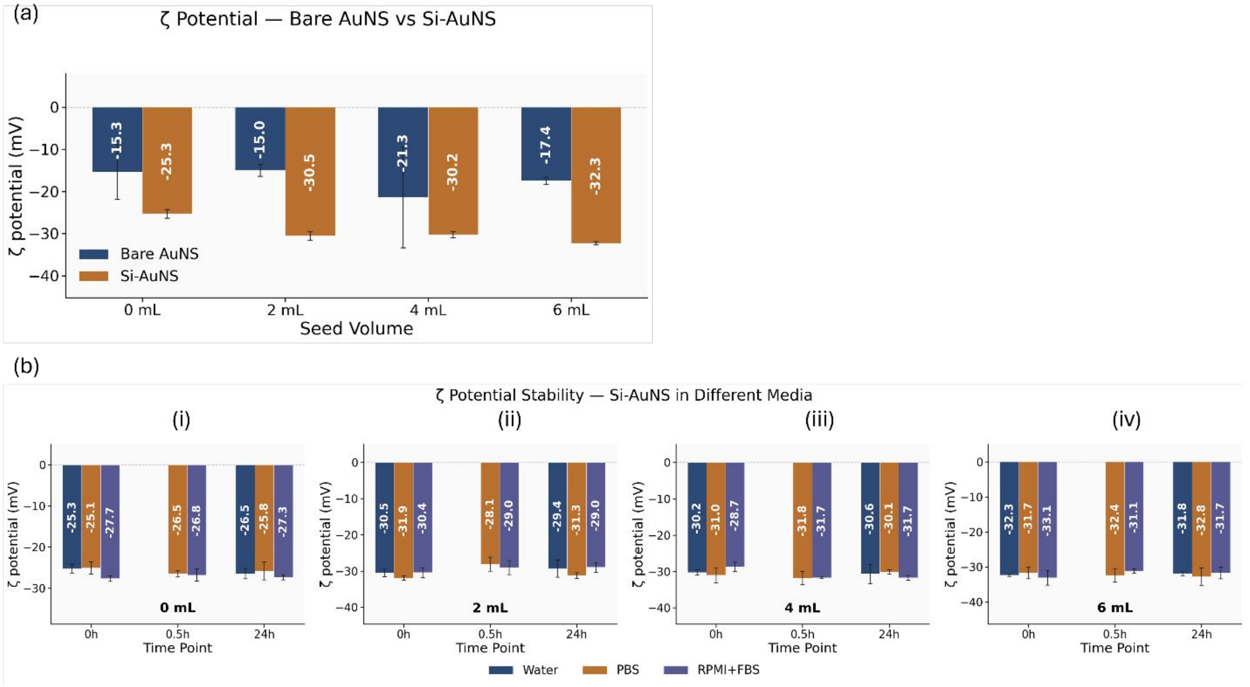

Figure S4. Zeta potential of bare and silica-coated AuNS before and during stability testing. (a) Zeta potential of bare AuNS and Si-AuNS across all four seed volume formulations. Silica coating consistently produced more negative surface charges than bare AuNS across all formulations, with zeta potential values of the Si-AuNS ranging from -25.3 mV to -32.3 mV. (b)(i--iv) Zeta potential stability of Si-AuNS in deionized water (dark blue), PBS (amber), and complete RPMI + FBS (purple) at 0 h, 0.5 h, and 24 h for the 0 mL, 2 mL, 4 mL, and 6 mL formulations respectively. All formulations maintained stable, moderately negative zeta potentials across all three media and time points, with minimal variation over ~24h, confirming electrostatic colloidal stability prior to cell exposure and establishing a pre-cell baseline for the surface charge evolution observed during macrophage interaction.

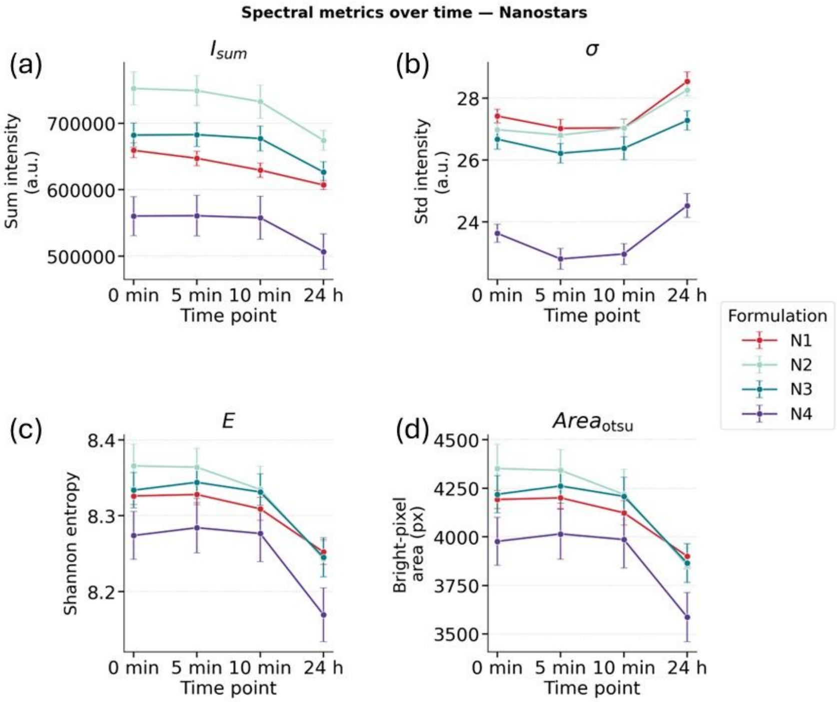

Figure S5. Temporal evolution of four hyperspectral feature metrics extracted from cross-correlated AuNS-only image stacks for N1–N4 nanostars interacting with THP-1-derived M0 macrophages. (a) Summed scattering intensity (I_sum), (b) standard deviation of intensity (σ), (c) Shannon entropy (E), and (d) Otsu-thresholded bright-pixel area (Area_otsu) are shown at 0 min, 5 min, 10 min, and 24 h post-exposure. Data points represent mean values; error bars indicate ± 1 standard deviation across all pixels within each condition.

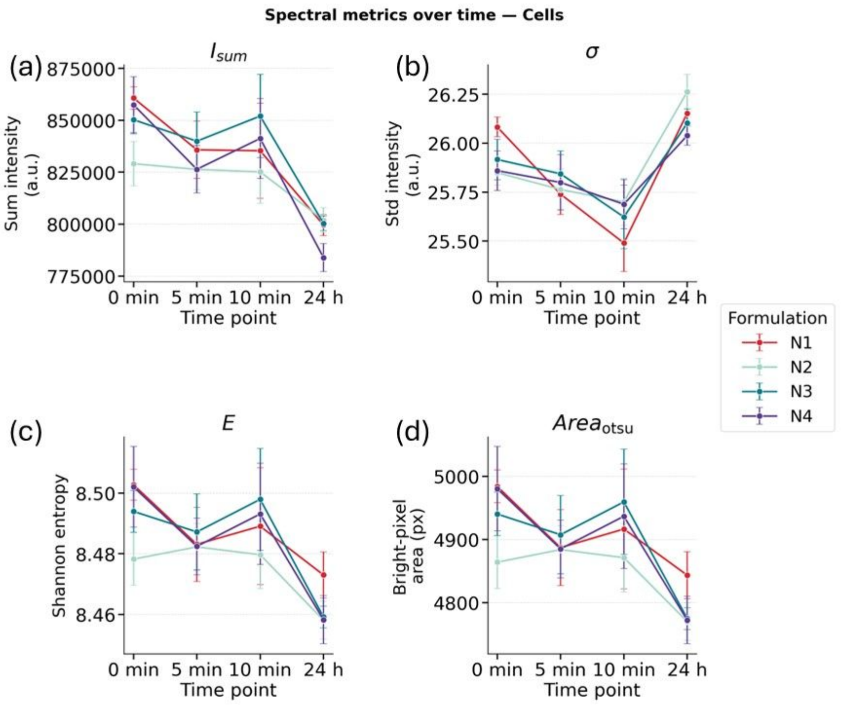

Figure S6. Temporal evolution of hyperspectral feature metrics extracted from cross-correlated cell-only image stacks for THP-1-derived M0 macrophages exposed to N1–N4 nanostar formulations. (a) I_sum, (b) σ, (c) E, and (d) Area_otsu are shown at 0 min, 5 min, 10 min, and 24 h. The near-identical trajectories across all four formulations indicate that macrophage spectral signatures are formulation-independent, confirming that formulation-specific trends observed in the nanostar-derived stacks (Figure S5) originate from nanoparticle optical evolution rather than cellular responses.

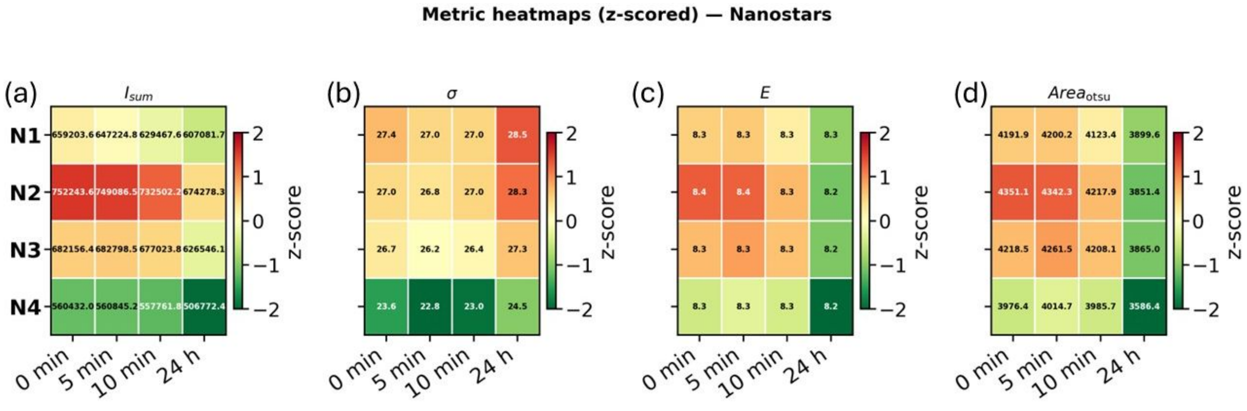

Figure S7. Heatmap visualization of z-scored spectral metrics for AuNS-only cross-correlated image stacks across four nanostar formulations (N1–N4, rows) and four time points (columns). Colors indicate the z-score relative to the global mean of each metric, with red denoting above-average and green denoting below-average values. Raw mean values are annotated within each cell. N4 consistently occupies the lowest z-score region across all metrics, indicating the smallest scattering intensity, heterogeneity, and bright-area fraction, consistent with its superior colloidal stability.

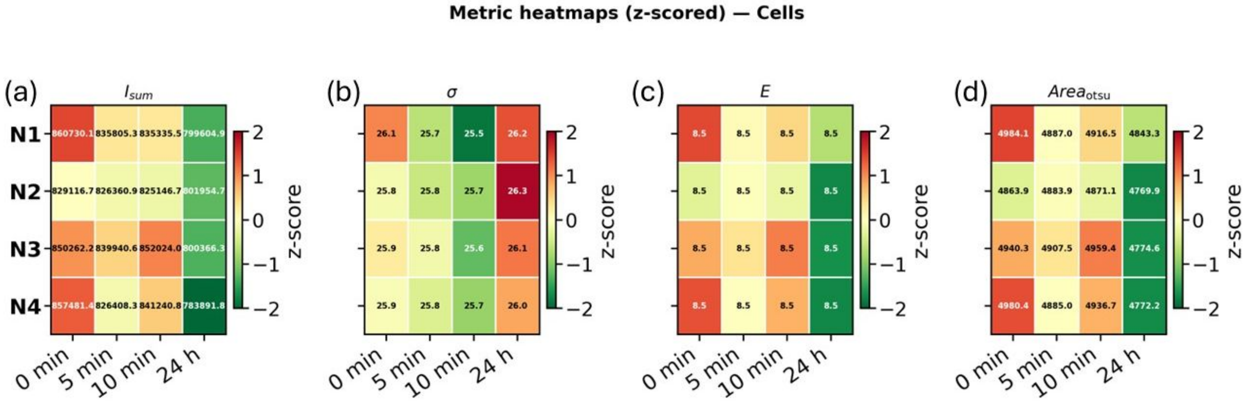

Figure S8. Heatmap visualization of z-scored spectral metrics extracted from cell-only cross-correlated image stacks. Unlike the nanostar-derived heatmaps (Figure S7), no systematic row-wise (formulation-dependent) pattern is apparent, and z-score differences across formulations are minimal. This confirms that macrophage spectral contributions do not introduce formulation-specific bias into the cross-correlation analysis.

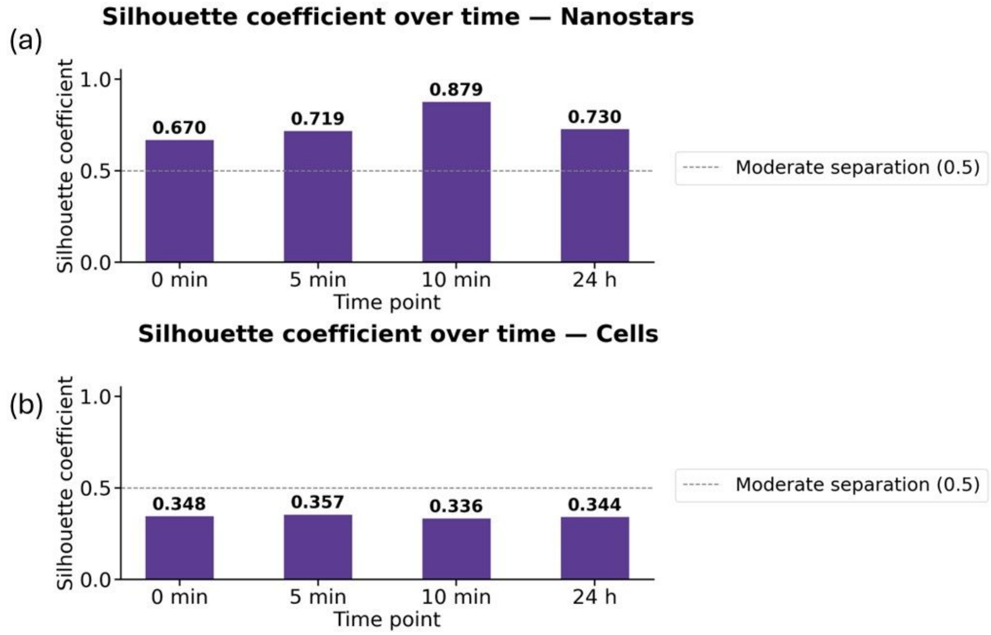

Figure S9. Silhouette coefficient of UMAP cluster embeddings at each time point for (a) AuNS-only and (b) cell-only cross-correlated image stacks. Values above 0.5 (dashed reference line) indicate moderate-to-strong cluster separation. Nanostar embeddings maintain silhouette scores of 0.67–0.88 throughout the experiment, reflecting well-separated, formulation-specific clusters. Cell embeddings remain consistently below 0.5 across all time points, confirming that cellular spectral contributions do not produce distinct formulation-dependent clustering in UMAP space.

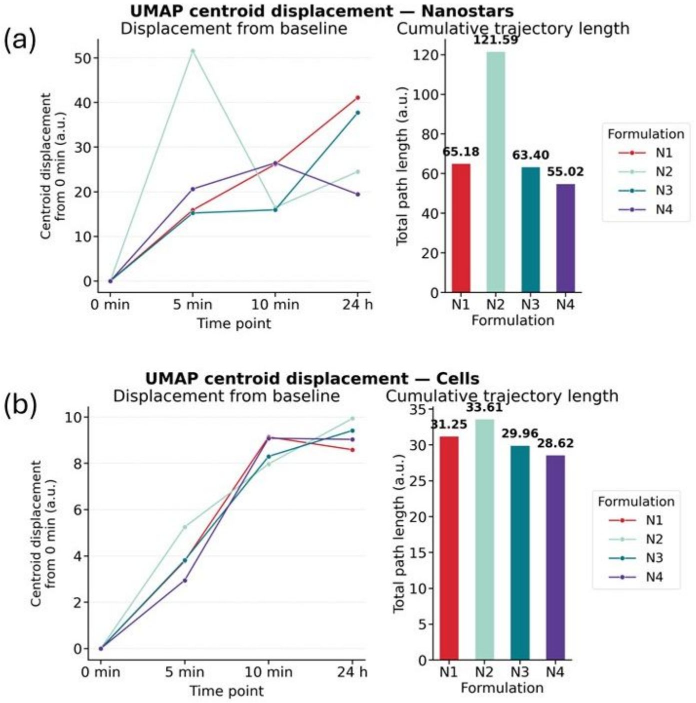

Figure S10. Centroid displacement analysis of UMAP embeddings for (a) AuNS-only and (b) cell-only cross-correlated image stacks. (a) Euclidean displacement of each formulation's UMAP centroid from its 0 min position, plotted over time. (b) Total cumulative path length traversed by each centroid across all four time points. For nanostars, N2 shows the largest path length (121.59 a.u.) driven by a transient spike at 5 min, while N4 exhibits the smallest cumulative displacement (55.02 a.u.), consistent with its superior optical stability. Cell embeddings show formulation-independent trajectories with uniformly low path lengths (28–34 a.u.), approximately half those of the nanostar embeddings.

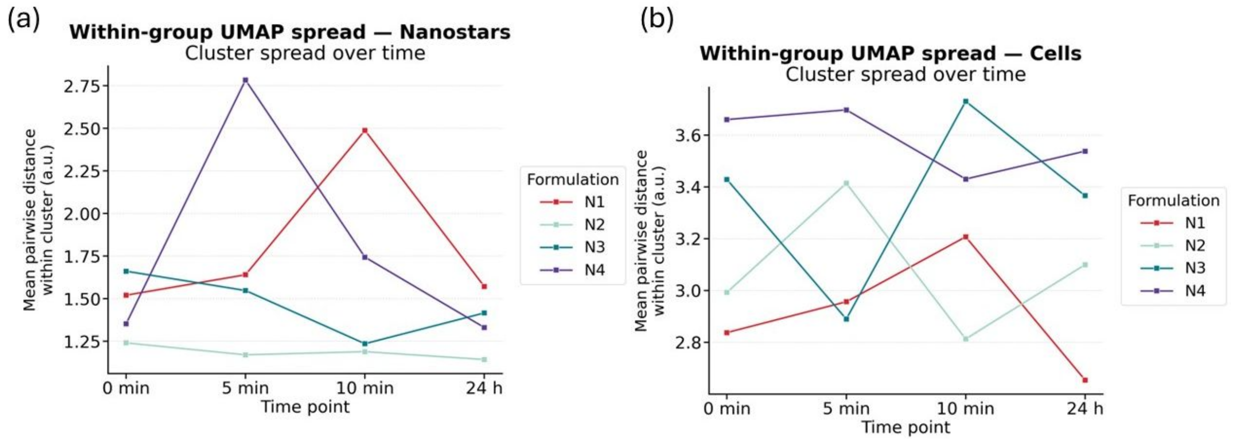

Figure S11. Mean pairwise distance within each formulation's UMAP cluster at each time point for (a) AuNS-only and (b) cell-only cross-correlated image stacks. A smaller mean pairwise distance indicates a tighter, more cohesive cluster. For nanostars, N2 and N4 display distinct spreading dynamics compared to N1 and N3, with N2 showing a transient increase at 5 min before contracting. Cell embeddings show broader and more variable within-group spread without consistent formulation-dependent ordering, further supporting the specificity of the nanostar-derived optical signatures.

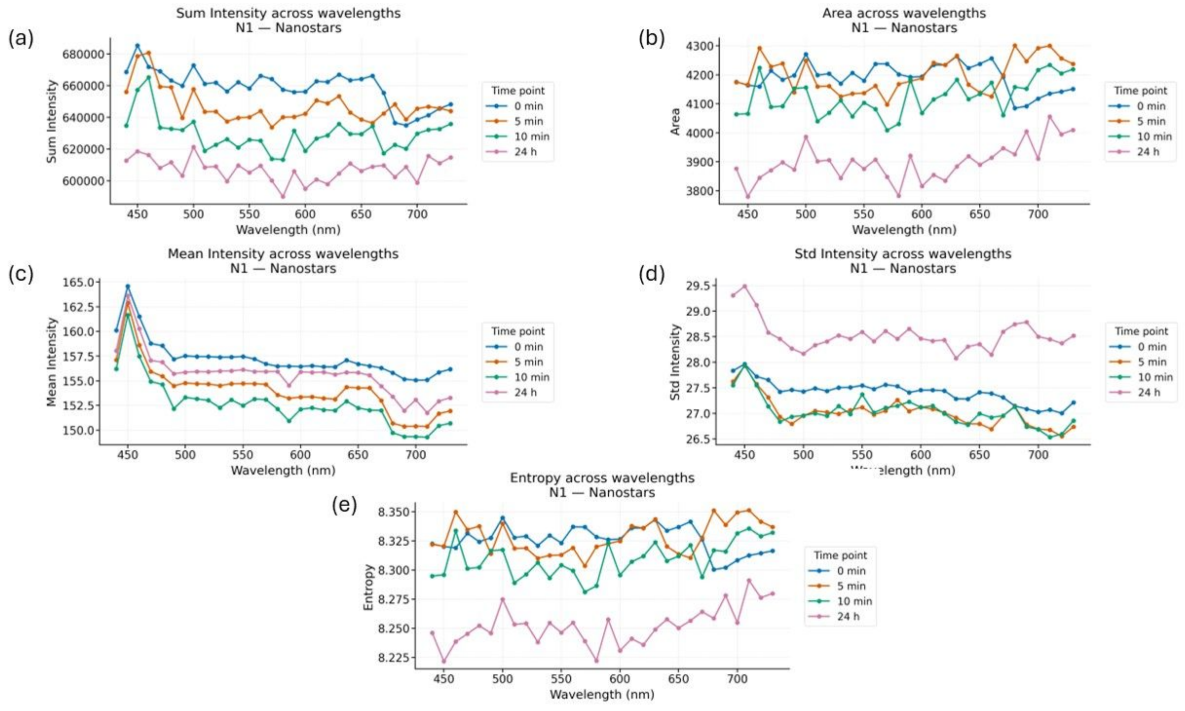

Figure S12. Wavelength-resolved hyperspectral metrics for N1 nanostars interacting with THP-1-derived M0 macrophages (cross correlated data for the nanostars). (a) Summed scattering intensity (I_sum), (b) Otsu-thresholded bright-pixel area (Area_otsu), (c) mean intensity, (d) standard deviation of intensity (σ), and (e) Shannon entropy (E) are shown as a function of wavelength across the 30 hyperspectral bands (440–730 nm) at four time points following AuNS exposure: 0 min (blue), 5 min (amber), 10 min (teal), and 24 h (pink).

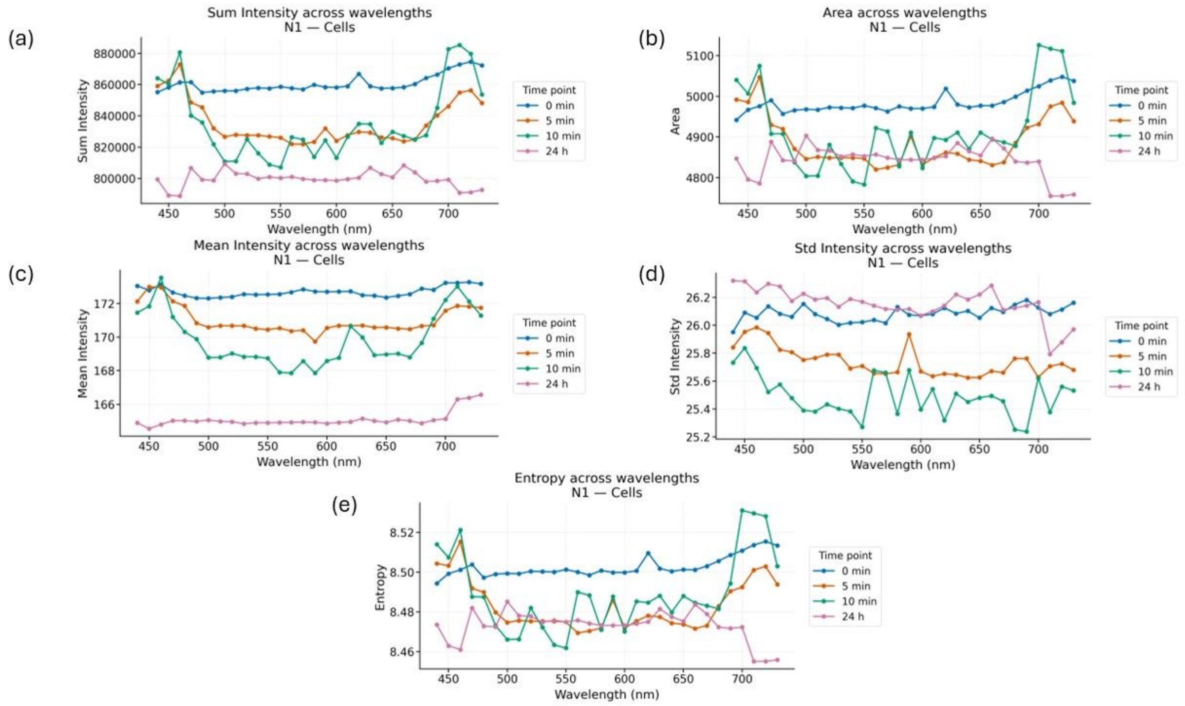

Figure S13. Wavelength-resolved hyperspectral metrics for N1 nanostars interacting with THP-1-derived M0 macrophages (cross correlated data for the cells). (a) Summed scattering intensity (I_sum), (b) Otsu-thresholded bright-pixel area (Area_otsu), (c) mean intensity, (d) standard deviation of intensity (σ), and (e) Shannon entropy (E) are shown as a function of wavelength across the 30 hyperspectral bands (440–730 nm) at four time points following AuNS exposure: 0 min (blue), 5 min (amber), 10 min (teal), and 24 h (pink).

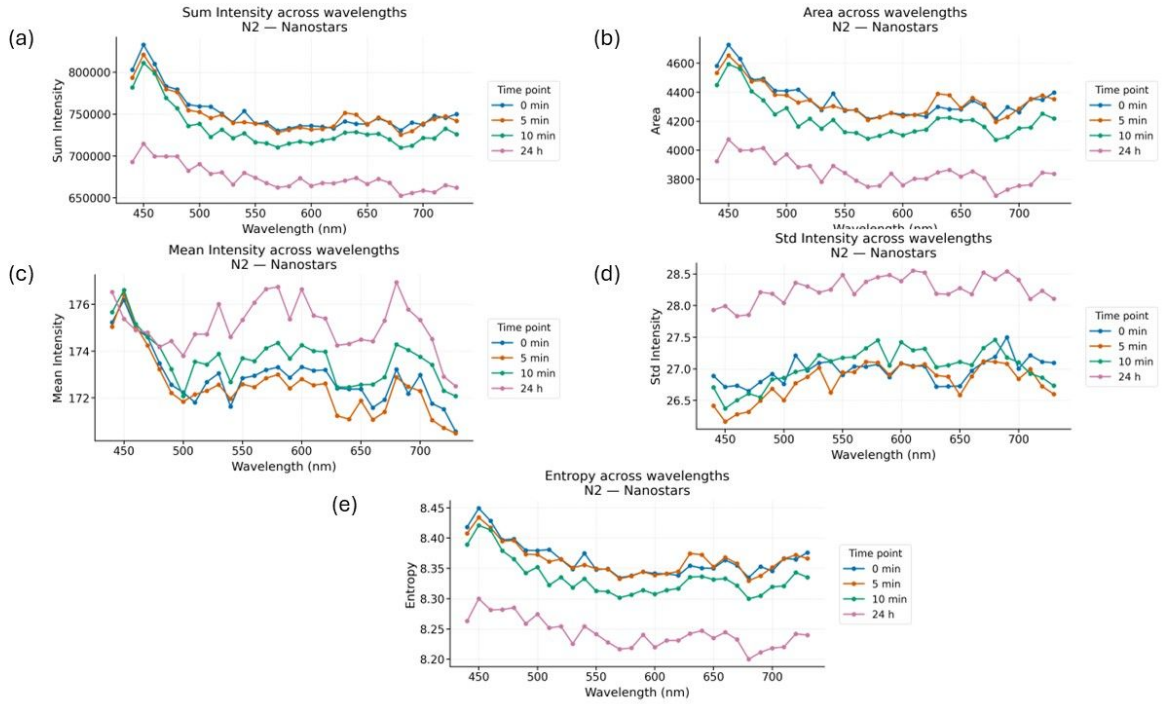

Figure S14. Wavelength-resolved hyperspectral metrics for N2 nanostars interacting with THP-1-derived M0 macrophages (cross correlated data for the nanostars). (a) Summed scattering intensity (I_sum), (b) Otsu-thresholded bright-pixel area (Area_otsu), (c) mean intensity, (d) standard deviation of intensity (σ), and (e) Shannon entropy (E) are shown as a function of wavelength across the 30 hyperspectral bands (440–730 nm) at four time points following AuNS exposure: 0 min (blue), 5 min (amber), 10 min (teal), and 24 h (pink).

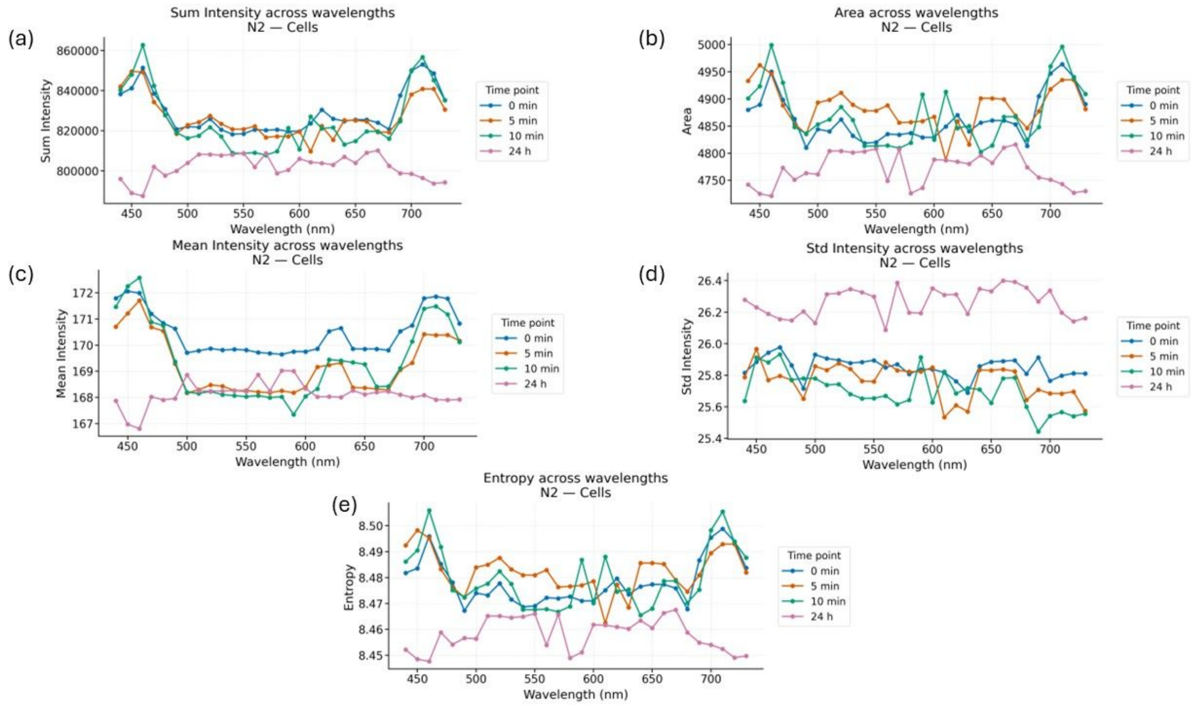

Figure S15. Wavelength-resolved hyperspectral metrics for N2 nanostars interacting with THP-1-derived M0 macrophages (cross correlated data for the cells). (a) Summed scattering intensity (I_sum), (b) Otsu-thresholded bright-pixel area (Area_otsu), (c) mean intensity, (d) standard deviation of intensity (σ), and (e) Shannon entropy (E) are shown as a function of wavelength across the 30 hyperspectral bands (440–730 nm) at four time points following AuNS exposure: 0 min (blue), 5 min (amber), 10 min (teal), and 24 h (pink).

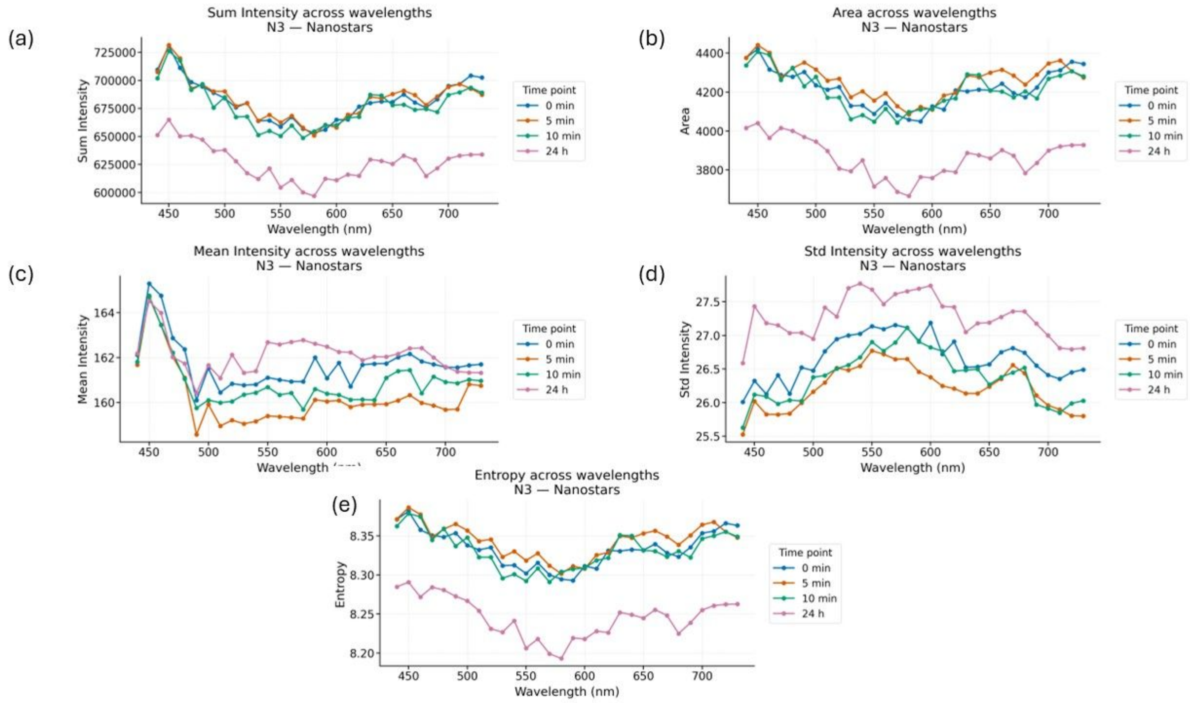

Figure S16. Wavelength-resolved hyperspectral metrics for N3 nanostars interacting with THP-1-derived M0 macrophages (cross correlated data for the nanostars). (a) Summed scattering intensity (I_sum), (b) Otsu-thresholded bright-pixel area (Area_otsu), (c) mean intensity, (d) standard deviation of intensity (σ), and (e) Shannon entropy (E) are shown as a function of wavelength across the 30 hyperspectral bands (440–730 nm) at four time points following AuNS exposure: 0 min (blue), 5 min (amber), 10 min (teal), and 24 h (pink).

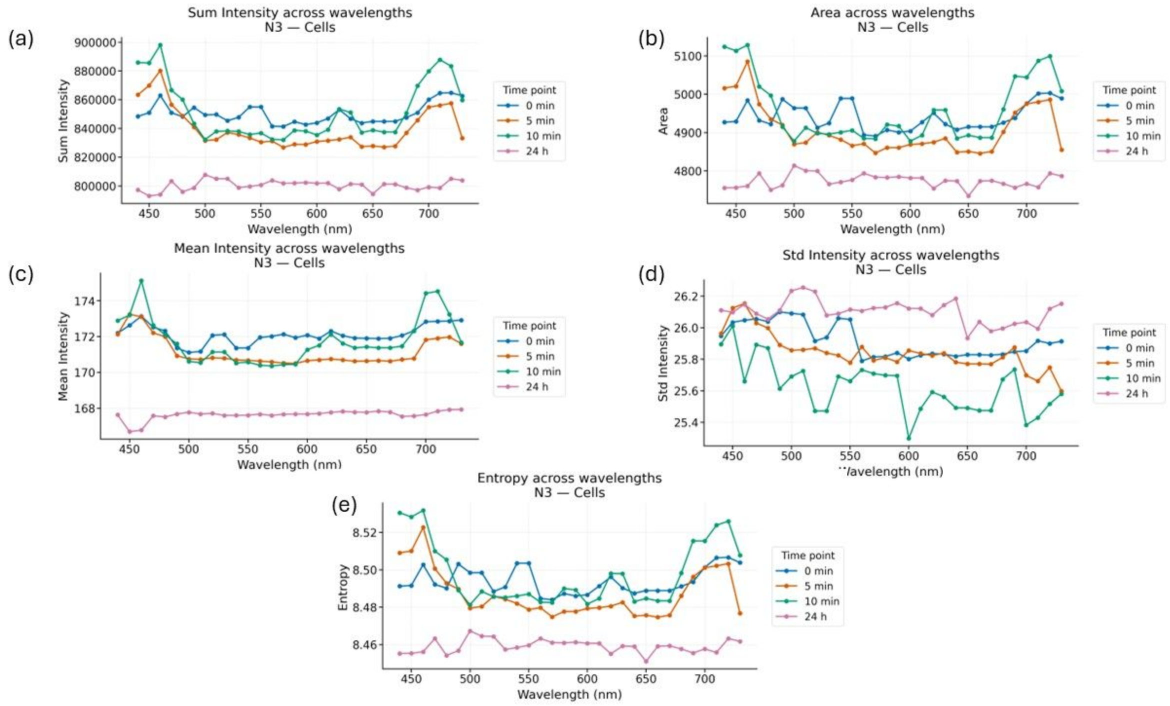

Figure S17. Wavelength-resolved hyperspectral metrics for N3 nanostars interacting with THP-1-derived M0 macrophages (cross correlated data for the cells). (a) Summed scattering intensity (I_sum), (b) Otsu-thresholded bright-pixel area (Area_otsu), (c) mean intensity, (d) standard deviation of intensity (σ), and (e) Shannon entropy (E) are shown as a function of wavelength across the 30 hyperspectral bands (440–730 nm) at four time points following AuNS exposure: 0 min (blue), 5 min (amber), 10 min (teal), and 24 h (pink).

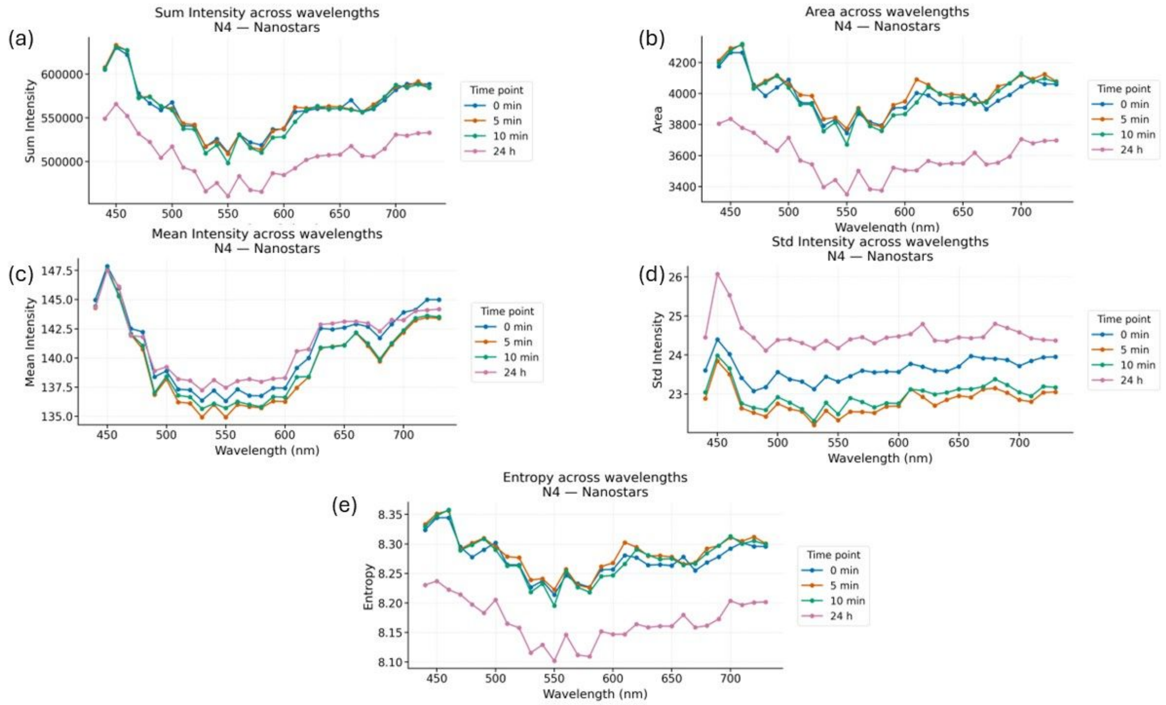

Figure S18. Wavelength-resolved hyperspectral metrics for N4 nanostars interacting with THP-1-derived M0 macrophages (cross correlated data for the nanostars). (a) Summed scattering intensity (I_sum), (b) Otsu-thresholded bright-pixel area (Area_otsu), (c) mean intensity, (d) standard deviation of intensity (σ), and (e) Shannon entropy (E) are shown as a function of wavelength across the 30 hyperspectral bands (440–730 nm) at four time points following AuNS exposure: 0 min (blue), 5 min (amber), 10 min (teal), and 24 h (pink).

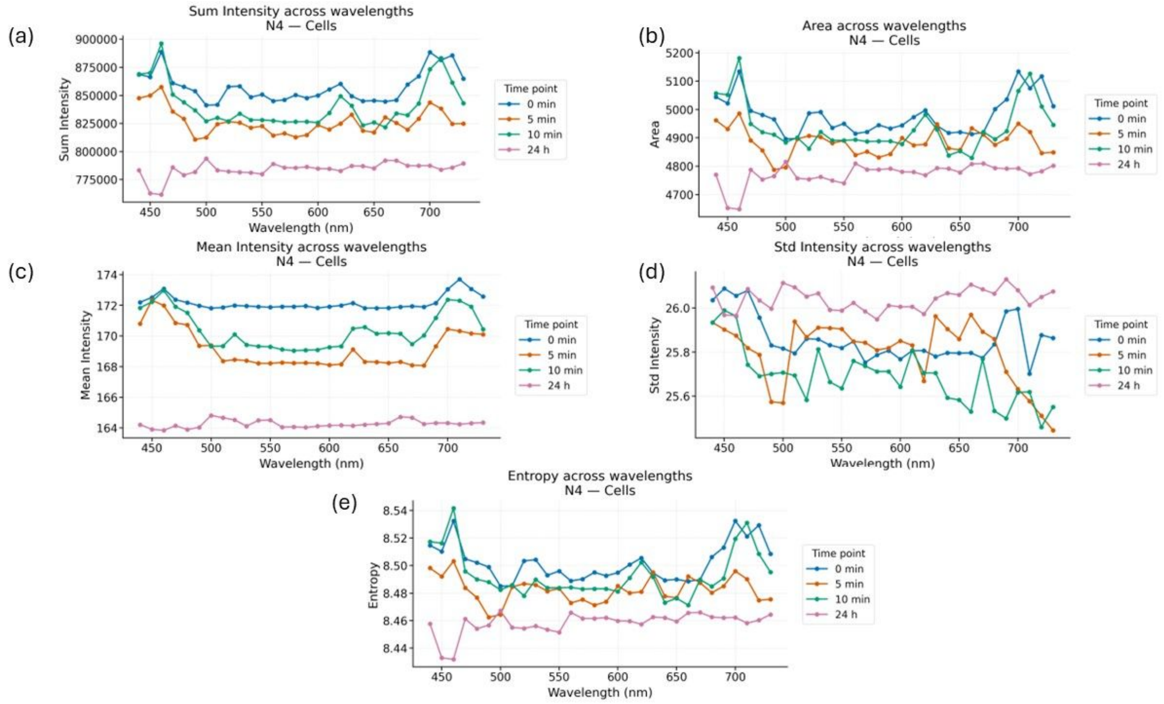

Figure S19. Wavelength-resolved hyperspectral metrics for N4 nanostars interacting with THP-1-derived M0 macrophages (cross correlated data for the cells). (a) Summed scattering intensity (I_sum), (b) Otsu-thresholded bright-pixel area (Area_otsu), (c) mean intensity, (d) standard deviation of intensity (σ), and (e) Shannon entropy (E) are shown as a function of wavelength across the 30 hyperspectral bands (440–730 nm) at four time points following AuNS exposure: 0 min (blue), 5 min (amber), 10 min (teal), and 24 h (pink).
